# Extractive single document summarization using binary differential evolution: Optimization of different sentence quality measures

**DOI:** 10.1371/journal.pone.0223477

**Published:** 2019-11-14

**Authors:** Naveen Saini, Sriparna Saha, Dhiraj Chakraborty, Pushpak Bhattacharyya

**Affiliations:** 1 Department of Computer Science and Engineering, Indian Institute of Technology Patna, Bihar, India; 2 Department of Computer Science and Application, University of North Bengal, Darjeeling, West Bengal, India; College of EME, NUST, PAKISTAN

## Abstract

With the increase in the amount of text information in different real-life applications, automatic text-summarization systems become more predominant in extracting relevant information. In the current study, we formulated the problem of extractive text-summarization as a binary optimization problem, and multi-objective binary differential evolution (DE) based optimization strategy is employed to solve this. The solutions of DE encode a possible subset of sentences to be present in the summary which is then evaluated based on some statistical features (objective functions) namely, the position of the sentence in the document, the similarity of a sentence with the title, length of the sentence, cohesion, readability, and coverage. These objective functions, measuring different aspects of summary, are optimized simultaneously using the search capability of DE. Some newly designed self-organizing map (SOM) based genetic operators are incorporated in the optimization process to improve the convergence. SOM generates a mating pool containing solutions and their neighborhoods. This mating pool takes part in the genetic operation (crossover and mutation) to create new solutions. To measure the similarity or dissimilarity between sentences, different existing measures like normalized Google distance, word mover distance, and cosine similarity are explored. For the purpose of evaluation, two standard summarization datasets namely, DUC2001, and DUC2002 are utilized, and the obtained results are compared with various supervised, unsupervised and optimization strategy based existing summarization techniques using ROUGE measures. Results illustrate the superiority of our approach in terms of convergence rate and ROUGE scores as compared to state-of-the-art methods. We have obtained 45% and 5% improvements over two recent state-of-the-art methods considering ROUGE−2 and ROUGE−1 scores, respectively, for the DUC2001 dataset. While for the DUC2002 dataset, improvements obtained by our approach are 20% and 5%, considering ROUGE−2 and ROUGE−1 scores, respectively. In addition to these standard datasets, CNN news dataset is also utilized to evaluate the efficacy of our proposed approach. It was also shown that the best performance not only depends on the objective functions used but also on the correct choice of similarity/dissimilarity measure between sentences.

## Introduction

Text summarization [1] is a natural language processing task which aims to create a summary describing the main theme of the document. Summarization techniques can be categorized into two groups depending on the extraction methodology: extractive [2] and abstractive [3–5]. In extractive summarization (ESDocSum), portions of the original document are used to form summary. While in abstractive summarization, reformulation of text is required which needs linguistic knowledge. Some of the application domains where text summarization techniques can be applied are web document summary generation, summarization of bug-reports, report generation and generation of personalized summary helping in question-answering systems. Because of the complexity of text documents and consideration of semantic and syntactic information present in texts, text-summarization has become a challenging natural language processing task.

Nowadays, sentence based extractive summarization [6–9] systems become popular where a set of sentences are extracted from the document for the overall understanding of a document. These set of sentences are selected using some sentence scoring features. Some such scoring features are position of the sentence in the document [9], the length of the sentence [9], similarity with respect to the title of document [9] etc.

In the existing literature, a lot of works have been reported to solve the summarization problem. Different learning paradigms have been tried like supervised [10], unsupervised [11], deep learning [12, 13], etc. But, in recent years, researchers have solved ESDocSum using different meta-heuristic optimization techniques like evolutionary algorithms which include genetic algorithm [14], differential evolution [15], etc. These algorithms help in extracting relevant sentences from the given document by optimizing some criteria. The algorithms have shown significant improvements [6, 9] over the existing methods. In this paper also, we have proposed a novel approach for single-document summarization which utilizes multi-objective binary differential evolution (MOBDE) [16] as the underlying optimization strategy. However, there exist several other optimization algorithms like AMOSA [17], PSO [18], etc. Several new concepts like self-organizing map [19] based mating pool generation etc. are introduced in our proposed framework. Before discussing the motivation behind developing such an algorithm, the existing works on ESDocSum are analyzed next.

### Related works

We have divided the related works on single document summarization into four categories: (a) supervised; (b) unsupervised; (c) meta-heuristic; and, (d) neural-network. Brief descriptions of these methods with the corresponding drawbacks are described below:

#### Supervised methods

SVM [20] considered pre-existing document-summary pair for learning. In [10], summarization problem is treated as a sequence labeling problem and is solved using *Condition Random Field* (CRF) [21]. In [22], a method named, *Manifold Ranking* was proposed in which a ranking score was assigned to each sentence in the document based on its information richness and diversity. Then, sentences having high ranking scores are only selected to generate the final summary. In [23], regression-based model was proposed using Integer Linear Programming [24] which uses three features to select the candidate summary from the set of available summaries. Main limitation of the methods proposed in these papers is that they make use of labeled data for training (i.e., whether sentence belongs to the summary or not) which requires manual effort and this is also a time-consuming step.

#### Un-supervised methods

In [25], QCS, a query-based method was proposed by Dunlavy et al. to generate the summary. It uses *Hidden Markov Model* (HMM) which predicts the probability of a sentence to be included in the summary or not. Note that the method developed was a graph-based method which was adopted for simultaneous summarization of single as well as multi-documents. Main drawback of this approach was that it considers only three features: sentence position, local saliency (for single-document summarization) and global saliency (for multi-document summarization) scores of the sentences. Ferreira et al. [8] developed a context-based summarization system and have shown that quality of generated summary obtained using different combinations (sum) of sentence scoring functions/features depends on the type of text (news, article, blog). Their sentence scoring features include word-based scoring (like term frequency, etc.), graph-based scoring (obtained using Text Rank algorithm [7]) and sentence-based scoring (sentence position, sentence similarity with the title, etc.). Main limitation of the discussed unsupervised methods [8, 25] is that they have not explored the feature like readability which is important in understanding the generated summary by the end-user.

#### Meta-heuristics based methods

Aliguliyev et al. [6] proposed an optimization based automatic text summarization method. Here, the sentences in the document are assigned to different clusters and clusters quality are optimized using differential evolution algorithm. Then in every cluster, sentences are sorted based on some sentence scoring features. Finally, high ranked sentences are selected as a part of the summary. In [26], fuzzy evolutionary optimization model (FEOM) was developed and applied to extractive summarization as an application. In [9], the method, *MA-SingleDocSum* was proposed by Mendoza et al. using optimization algorithm named as *Memetic algorithm*. It makes use of guided local search to solve the summarization problem. In [27], a method named ESDS-GHS-GLO is proposed based on Global-best Harmony Search meta-heuristic and a greedy local search procedure. It considers extractive single document summarization as a binary optimization problem. Rasim et al. [28] proposed a COSUM method utilizing clustering and optimization technique optimizing coverage and diversity of the summary simultaneously. Main drawbacks of these meta-heuristic algorithms are their low convergence rate and low ROUGE score. Moreover, they optimized sum (in some of the cases, weighted sum) of different objective functions, thus, converting multiple objective values to a single value.

#### Neural-network based methods

In [11], a neural network based method was developed namely, NetSum, which uses the RankNet [29] algorithm to assign a rank to the sentences in the document and then identifies informative sentences. In recent years [12, 13], some deep learning models like a recurrent neural network, etc. have been used for solving single document extractive summarization task. Note that these methods make use of supervised information while training.

### Motivation

Existing meta-heuristic strategies suffer from the following problems: slow convergence and low ROUGE score values for the obtained summary. None of the existing approaches makes use of the self-organizing map (SOM) in their architecture, which can help in exploring the neighborhood of a solution in an efficient way to determine the optimal ROUGE score. Here, ROUGE score is an evaluation function to measure the informativeness of the summary. In the current paper, summarization problem is treated as a binary optimization problem where different quality measures of the summary are optimized simultaneously. Six objective functions, namely, the position of the sentence in the document, the similarity of a sentence with the title, length of the sentence, cohesion, readability, coverage are selected to be optimized simultaneously. Multi-objective binary differential evolution (MOBDE) [16] is used as the underlying optimization strategy to optimize all objective functions simultaneously where each chromosome (or solution) is a binary string representing a set of possible sentences to be selected in the generated summary. Optimization of multiple objective functions helps in generation of good quality summary for a given document and thus, attaining better ROUGE score.

To increase the convergence rate further, concepts of self-organizing map (SOM) [19, 30] are incorporated in MOBDE framework. SOM is a type of neural network which maps high dimensional input space to low-dimensional output space, where, output space is a grid of neurons arranged in 2-dimensional space. The central principle behind the SOM is that input samples which are close to each other in the input space should also come close to each other in the output space. Thus, it can be used as a cluster analysis tool. In any evolutionary algorithm, the qualities of new solutions generated from the old solutions play vital roles in convergence as they help in reaching the global optimum solutions. In our approach, SOM is used to generate high-quality solutions which in turn help in faster convergence. SOM is first trained using the current population to discover the localities of chromosomes/solutions, and then a mating pool is constructed for each chromosome using the neighborhood relationships extracted by SOM. After that, chromosomes present in the mating pool are combined using reproduction operators (crossover and mutation) [31] to generate some new solutions.

#### The reason behind using MOBDE

MOBDE shows superior performance [32–34] over the existing evolutionary algorithms like NSGA-II [14], MOEA/D [35], etc. Moreover, researchers have shown effectiveness of DE in solving different real-life optimization problems like clustering [36–41], summarization [42–44]. It was shown in the literature that DE also outperforms [18, 45, 46] Particle Swarm Optimization (PSO) [47] which is another optimization strategy.

To show that the performance of the proposed summarization technique not only depends on objective functions considered but, also on the type of sentence similarity/dissimilarity function used, experiments are conducted by varying the similarity/dissimilarity measures, namely normalized Google Distance (NGD) [48], word mover distance [49] and cosine similarity [6]. The proposed approach is tested on two standard datasets of text summarization namely, DUC2001 and DUC2002 (https://www-nlpir.nist.gov/projects/duc/data.html). One more dataset related to CNN news [23] is also used to evaluate the efficacy of our proposed system. Results obtained clearly show the superiority of our proposed algorithm in comparison to various state-of-the-art techniques.

### Contributions

The major contributions of this paper are enumerated below:

- In the literature, ESDocSum problem is often formulated as a single objective optimization problem with the weighted sum of different objectives [6, 9] and this is popularly solved using different EA techniques. However, in this paper, summarization problem is treated as a multi-objective optimization problem where various aspects of summary like the readability of the summary, the similarity of the sentences in the summary with the title, etc. are optimized simultaneously.
- In the existing multi-objective evolutionary algorithms, usually, reproduction operators like roulette wheel selection, tournament selection [14] etc., popularly used in a single-objective optimization framework, are used to generate new solutions. But, in the current study, to generate high-quality solutions, some newly designed self-organizing map based genetic operators are used which further help in reaching the global optimum solutions in a faster way.
- In order to show that performance of summarization system not only depends on the objective functions used but also depends on the type of similarity/dissimilarity measure used between sentences, three types of similarity/dissimilarity measures namely, normalized google distance [48], word mover distance [49], and cosine similarity [6], are explored in this paper.
- Most of the papers on summarization using some optimization strategies make use of actual summary to report the results. But, in real time situations, actual summary may not be available. Therefore, in this paper, we have explored various unsupervised strategies for selecting a single solution from the final Pareto optimal front produced by any multi-objective optimization based technique.

## Background knowledge

This section discusses some related concepts used in our proposed framework.

### Multi-objective optimization

Multi-objective optimization (MOO) [14] refers to the task of optimizing more then one objective function, simultaneously, to solve a particular problem. It provides a set of alternative solutions to the decision maker as oppose to the single objective optimization. Mathematically, MOO can be formulated as:

$$\begin{array}{c} \text{max}\left\{f_{1}\left(\vec{x}\right),f_{2}\left(\vec{x}\right)\ldots f_{m}\left(\vec{x}\right)\right\}\;\text{such}\;\text{that}\;\vec{x}\in X \end{array}$$ (1)

where *X* is a set of decision vectors in *n*-dimensional space denoted as $\left\{\vec{x_{1}},\vec{x_{2}}\ldots \vec{x_{n}}\right\}$, *m* is the number of objective functions to be maximized and should be grater than 2. Note that there can be some constraints as a part of the optimization process.

### A binary differential evolutionary algorithm for optimization

Differential Evolution (DE) is a population-based global optimization technique proposed by Storn and Price [15] to solve real-world problems. There exist many variants of the DE; each differs in representation (real-coded or binary-coded) of the solution and in the use of parameters. In our paper, a binary differential evolution algorithm [16] is used where each solution is represented as a binary vector. Each solution is associated with two or more objective functions in DE framework for multi-objective optimization. It executes similar to any other evolutionary algorithms. It starts with a set of solutions called as population. At time stamp (generation) t, *ith* solution is represented as

$$\begin{array}{c} {\vec{x}}_{i}\left(t\right)=\left[x_{i,1}\left(t\right),x_{i,2}\left(t\right),\ldots ,x_{i,n}\left(t\right)\right] \end{array}$$ (2)

where, *n* is the length of the solution and *i* = 1, 2…, ∣*P*∣, ∣*P*∣ is the size of the population, *x*_*i*,*m*_ can take value either 0 or 1 for *m* = 1, 2…, *n*. For each current solution *i*, offspring *y*′ is generated using crossover and mutation operations [15, 50] and then it undergoes evaluation in comparison with current solution, *i*. Crossover is the exchange of components between two solutions and mutation is the modification in the component. Only the better solution in terms of objective function value out of these two solutions (current and new offspring) can survive into the next generation.

## Problem definition

Consider a document *D* consisting of *N* sentences, {*s*_1_, *s*_2_, …, *s*_*N*_}. Our main task is to find a subset of sentences, *S* ∈ *D*, such that

$$\begin{array}{c} \sum_{s_{i}\in S}l_{i}\leq S_{max} \end{array}$$ (3)

where, *S* represents the main theme/topic of the document or subset of sentences which cover the relevant information from the document, *s*_*i*_ is the sentence belonging to *S*, *l*_*i*_ measures the length of *ith* sentence in terms of number of words, *S*_*max*_ is the maximum number of words allowed in generated summary.

## Sentence similarity/dissimilarity measures and sentence scoring features

To select the best possible set of sentences to be present in the summary, various statistical features (fitness functions or objective functions) are used to evaluate the subset and those are optimized simultaneously using the binary differential evolution algorithmic framework. Some of the features use similarity/dissimilarity criteria between sentences. In the current paper, we have utilized different types of similarity/dissimilarity measures and statistical functions. Descriptions of these functions/features are given below:

### Sentence similarity/dissimilarity measures

In our work, three similarity/dissimilarity measures are used: Normalized Google Distance [48], word mover distance [51] and cosine similarity [6].

#### Normalized google distance

Normalized Google Distance (NGD) measures the semantic relationship between two sentences using terms present in the sentences. It was first proposed in [6]. Two terms tend to be close to each other if they are having similar sense. It is important to note that it is a dissimilarity measure, not a distance function. NGD between two sentences, *s*_*i*_ and *s*_*j*_, can be defined as:

$$\begin{array}{c} d_{NGD}\left(s_{i},s_{j}\right)=\frac{\sum_{t_{1}\in s_{i}}\sum_{t_{2}\in s_{j}}NGD\left(t_{1},t_{2}\right)}{nt_{i}\times nt_{j}} \end{array}$$ (4)

where, *t*_1_ and *t*_2_ are the terms belonging to sentences, *s*_*i*_ and *s*_*j*_, respectively; *nt*_*i*_ and *nt*_*j*_ are the number of terms in sentence *s*_*i*_ and *s*_*j*_, respectively; NGD can be expressed as:

$$\begin{array}{c} NGD\left(t_{1},t_{2}\right)=\frac{\text{max}\left\{\text{log}\left(f_{t1}\right),\;\text{log}\left(f_{t2}\right)\right\}-\text{log}\left(f_{t1,t2}\right)}{\text{log}\;N-\text{min}\{\text{log}\left(f_{t1}\right),\;\text{log}\left(f_{t2}\right)\}} \end{array}$$ (5)

where, *f*_*t*1_ denotes the number of sentences in the document (*D*) containing term *t*1, *f*_*t*2_ denotes the number of sentences in the document (*D*) containing term *t*2, *f*_*t*1,*t*2_ indicates the number of sentences in the document (*D*) containing both terms, *t*1 and *t*2, *N* is the number of sentences in the document. Three important properties of NGD are listed below:

1. The range of *d*_*NGD*_(*s*_*i*_, *s*_*j*_) lies in the scale of 0 to ∞.
2. If *t*_1_ = *t*_2_ or if *t*_1_ ≠ *t*_2_, but *f*_*t*1_ = *f*_*t*2_ = *f*_*t*1,*t*2_ > 0, then *d*_*NGD*_(*s*_*i*_, *s*_*j*_) = 0
3. For every sentence *s*_*i*_, *d*_*NGD*_(*s*_*i*_, *s*_*i*_) = 0.

Note that if *N* = 1, then we have *f*_*t*1_ = *f*_*t*2_ = *f*_*t*1,*t*2_. In this case, $d_{NGD}\left(t1,\;t2\right)=\frac{0}{0}$, will be considered as 0 by the 2*nd* property of NGD.

#### Word mover distance

Word Mover Distance (WMD) [49, 51, 52] calculates the dissimilarity between two texts as the amount of distance that the *embedded words* [53] *of one text needs to travel to reach the embedded words of another text* [51]. In our approach, text means a sentence. To obtain word embedding of different words, it makes use of word2vec [53]. If two sentences are similar, then WMD will be 0.

#### Cosine similarity

Cosine similarity [6] is a measure of similarity between two non-zero vectors that measures the cosine of the angle between vectors. It can be defined as:

$$\begin{array}{c} \text{cos}\left(\theta \right)=\frac{\vec{V_{1}}.\vec{V_{2}}}{\left\|\vec{V_{1}}\|\|\vec{V_{2}}\right\|}=\frac{\sum_{i=1}^{n}V_{1_{i}}V_{2_{i}}}{\sqrt{\sum_{i=1}^{n}V_{1_{i}}}\sqrt{\sum_{i=1}^{n}V_{2_{i}}}} \end{array}$$ (6)

where, $\vec{V_{1}}$ and $\vec{V_{2}}$ are the vectors of length *n*, $V_{j_{i}}$ is the *ith* component of *jth* vector, *j* = 1, 2.

The value of this similarity lies between -1 to 1. 1 means two vectors are overlapping or exactly similar to each other, -1 means two vectors are opposite to each other, and 0 indicates they are orthogonal to each other. As our documents contain texts, in order to measure cosine similarity between two sentences, sentence vectors are required. For this purpose, word2vec [53] tool is used.

Word2vec [53] is a model that is used to generate word embedding. It is a two-layered neural network which takes a large corpus of text as the input and generates a unique vector of several hundred dimensions for each word in the corpus as the output. The main goal is to predict a word given the other words in a context. Therefore, it is capable of capturing the semantics between the two words. In our framework, pre-trained *word2vec* model on *googlenews* corpus (https://github.com/mmihaltz/word2vec-GoogleNews-vectors) is used. The sentence vector is obtained by averaging the word vectors of the words (obtained from the pre-trained word2vec model) present in the sentence.

### Statistical features or objective functions

To obtain a good summary, selection of objective functions (quality functions on sentences) is crucial. These objective functions assign some fitness values to the sentences and further help in improving the quality of generated summary. The set of objective functions used in our approach are: the position of the sentence in the document, similarity of a sentence with the title, length of the sentence, cohesion, coverage, and readability. First five objective functions are selected motivated by the paper [9]. Authors of cited paper have optimized *weighted sum* of first five objective functions and shown that their results are better that state-of-the-art results. But combining the values of different objective functions using weighted criteria into a single value may not be meaningful [54]. Moreover, in any text-based summarization system, readability is an important factor as generated summary should be readable to end-users. Therefore, in our approach, readability feature is considered as a sixth objective function. All these objective functions have to be maximized simultaneously by the use of some multi-objective optimization framework instead of using weighted sum approach. Brief description on these objective functions are provided below:

#### Sentence position

In any document, regardless of domain, relevant/informative sentences can be found in some sections of the document like the leading paragraph of the document. Therefore, to consider this information into account, sentence position [55, 56] is expressed as:

$$p=\sum_{\forall s_{i}\in Summary}\sqrt{(}\frac{1}{q_{i}})$$ (7)

where *q*_*i*_ is the position of *ith* sentence. It assigns higher scores to initial sentences of the document. As the sentence position in the document increases, the value of *p* decreases.

#### Similarity with title

Sentences in the summary should be similar to the title [57] to obtain a good summary because the title describes the theme of the document. This objective function is defined as given below:

$$\begin{array}{c} SWT_{avg}=\sum_{\forall s_{i}\in Summary}\frac{sim(s_{i},title)}{O}, \end{array}$$ (8)

$$\begin{array}{c} SWT=\frac{SWT_{avg}}{max_{\forall Summary}SWT} \end{array}$$ (9)

where, *title* is the headline/title of the document in which sentence *s*_*i*_ belongs to, *sim*(*s*_*i*_, *s*_*j*_) is the similarity between sentences, *s*_*i*_ and *s*_*j*_, O is the number of sentences in generated summary, *SWT*_*avg*_ is the average similarity of the sentences in summary with the title, *max*_∀*Summary*_ *SWT* is the average maximum similarities of all sentences with the title, and *SWT* is the similarity factor of the summary *S* with the title. SWT is close to 1 if sentences, in summary, are closely related to the title of the document.

#### Sentence length

Literature survey suggests that shorter sentences have less chances to appear as a part of the summary [58]. In this paper, normalization based sigmoid function [59] is used which favors the longest sentence but does not entirely rule out the medium length sentences. Mathematically, it is expressed as:

$$\begin{array}{c} \sum_{\forall s_{i}\in Summary}\frac{\left(1-\text{exp}(\frac{-l\left(s_{i}\right)-\mu \left(l\right)}{std(l)})\right)}{\left(1+\text{exp}(\frac{-l\left(s_{i}\right)-\mu \left(l\right)}{std(l)})\right)} \end{array}$$ (10)

Where, *μ*(*l*) is the mean length of sentences in the summary, *l*(*s*_*i*_) is the length of sentence, *s*_*i*_, and *std*(*l*) is the standard deviation of lengths of sentences in summary *S*.

#### Cohesion

Cohesion [60, 61] measures the relatedness of the sentences in the summary. For a good summary, relatedness between sentences should be tightly coupled. It is expressed as

$$\begin{array}{c} COH=\frac{\text{log}(C_{s}\times 9+1)}{\text{log}(M*9+1)} \end{array}$$ (11)

Where,

$$\begin{array}{c} C_{s}=\frac{\sum_{\forall s_{i},s_{j}\in Summary}sim\left(s_{i},s_{j}\right)}{O_{s}}\text{and,}\;O_{s}=\frac{N\times (N-1)}{2} \end{array}$$ (12)

*M* = max *sim*(*s*_*i*_, *s*_*j*_), *i*, *j* ≤ *N*, *C*_*s*_ measures the average similarity of the sentences in the summary, *sim*(*s*_*i*_, *s*_*j*_) is the similarity between sentences, *s*_*i*_ and *s*_*j*_, N is the total number of sentences in the document, M is the maximum similarity between two sentences. It ranges between [0, 1]. 1 indicates sentences in summary are highly related to each other.

#### Coverage

Coverage (CoV) [9] measures the extent to which sentences in the summary provide useful information about the document and should be maximized. Coverage is defined as

$$\begin{array}{c} CoV=\sum_{\forall s_{i}\in Summary}\sum_{\forall s_{j}\in Doc,s_{i}\neq s_{j}}\frac{sim(s_{i},s_{j})}{N-1} \end{array}$$ (13)

where *s*_*i*_ and *s*_*j*_ are the sentences belonging to generated summary and document, respectively, *Doc* is the document, *N* is the number of sentences in the document, *sim*(*s*_*i*_, *s*_*j*_) is the similarity between sentences, *s*_*i*_ and *s*_*j*_.

#### Readability factor

Readability factor [60] is the last objective function which is the most important factor for summary formation. In this, each sentence should be related to the previous one to make the summary readable. It is expressed as:

$$\begin{array}{c} R=\sum_{i=2}^{N_{p}}sim\left(s_{i},s_{i-1}\right) \end{array}$$ (14)

where, *N*_*p*_ is the number of sentences in the predicted summary, *s*_*i*_ and *s*_*i*−1_ are two consecutive sentences in the predicted summary, *sim*(*s*_*i*_, *s*_*i*−1_) is the similarity between sentences, *s*_*i*_ and *s*_*i*−1_.

## Self-organized multi-objective differential evolution based ESDocSum approach

**Fig 1:**
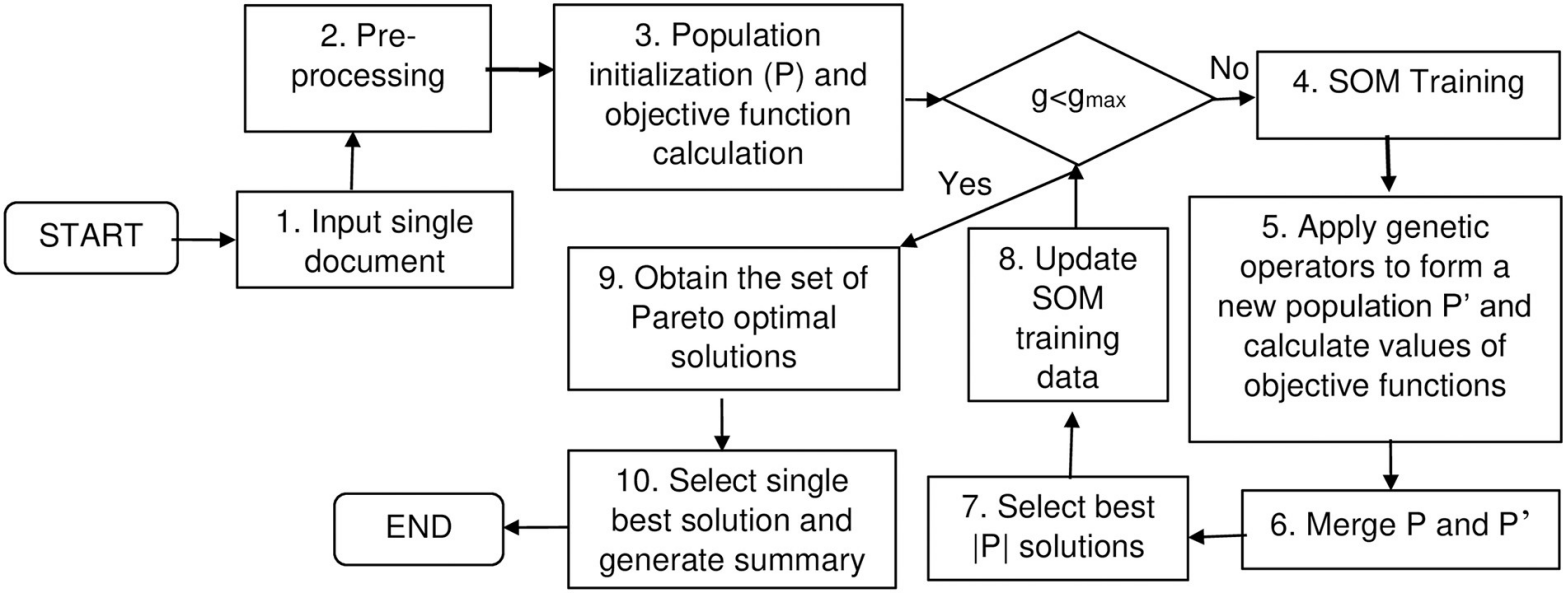
Proposed architecture. Where *g* is the current generation number initialized with 0; *g*_*max*_ is the maximum number of generations which is defined by the user; |*P*| is the number of solutions in the population. After step-8, *g* is incremented by 1 and the process continues until maximum number of generations is reached.

In this paper, two approaches were developed for sentence based extractive single document summarization. Note that both approaches utilize a multi-objective based differential evolution technique as the underlying optimization strategy. SOM-based genetic operators are introduced in the process to increase the convergence. The flowchart of the proposed approach is shown in Fig 1 and underlying steps are discussed in subsequent sections. The pseudo code is also provided in Algorithm 1.

1. Approach-1: In this approach all objective functions are assigned some importance factors/weights. For example, if fitness values of six objective functions are < *ob*_1_, *ob*_2_, *ob*_3_, *ob*_4_, *ob*_5_, *ob*_6_ > and weights assigned are < *α*, *β*, *γ*, *δ*, λ, *ϕ* >, then < *ob*_1_ × *α*, *ob*_2_ × *β*, *ob*_3_ × *γ*, *ob*_4_ × *δ*, *ob*_5_ × λ, *ob*_6_ × *ϕ* > are optimized simultaneously. The values of these weights are selected after conducting a thorough literature survey [9, 16, 62].
2. Approach-2: In this approach all objective functions are simultaneously optimized without assigning any weight values.

In the literature [9, 62], it was shown that some of the objective functions used in our approach have more importance than others. Therefore, Approach-1 is developed to see the effect of the varying importance of different objectives functions.

**Algorithm 1**: SOM-based Extractive Text Summarization

 **Data**: Single Text Document

 **Result**: The best solution and corresponding summary generated

1 Initialize population size |*P*|, population P (including calculation of objective functions) and *max*_*generation*;

2 Initialize training data for SOM as S = P;

3 t = 1;

4 **while** *t<max_generation* **do**

5  P′ ← [ ] //store new solutions;

6  Perform training of SOM using S;

7  **for** *each solution in P* **do**

8   Construct Mating pool (Q);

9   Generate new solution using Q, crossover and mutation;

10   Calculate new solution’s objective functional values;

11   Add new solution into P′;

12  **end**

13  P″ = Merge populations P and P′;

14  P ← Apply non-dominated sorting and crowding distance operator (if needed) on P″ to select the top ∣*P*∣ solutions;

15  Update SOM training data as S = P′\ P″;

16  t = t+1;

17 **end**

18 *return* the best solution from the final Pareto optimal front and corresponding summary;

### Preprocessing

Before generating the summary, a series of steps are executed to pre-process the document. These steps include segmentation of the document into sentences, stop word removal (frequent words like is, am, are, etc. are removed from the document), case folding (lower case conversion) and removal of punctuation marks. Here, the nltk toolkit [63] is used for document segmentation and removal of stop words.

### Representation of solution and population initialization

Any evolutionary algorithm, starts with a set of solutions (or chromosomes), $<{\vec{x}}^{1},{\vec{x}}^{2}\ldots {\vec{x}}^{|P|}>$, called as population, where, ∣*P*∣ is the number of solutions. As our approach is based on binary optimization, each solution is represented in the form of a binary vector. The size of the solution is set equal to the number of sentences in the document. For example, if a document consists of 10 sentences then a valid solution can be represented as [1, 0, 0, 1, 1, 0, 1, 0, 0, 0]. This solution indicates that first, fourth, fifth, and seventh sentences of the original document should be in the summary. The initial population is generated randomly. While generating the solution, the constraint on summary length is taken into account as $\sum_{s_{i}\in Summary}l_{i}\leq S_{max}$, where, *l*_*i*_ measures the length of sentence in terms of the number of words, *S*_*max*_ is the maximum number of words allowed in the generated summary.

### Objective functions used

To measure the quality of each solution in the population, a set of objective/fitness functions are evaluated. These functions are discussed in the previous section, and all are of maximization type. Note that optimization of these functions helps in getting a good quality summary.

### SOM training

In this step, SOM [19, 30] will be trained using the solutions in the population. In this paper, we have used the sequential learning algorithm to train the SOM. Readers can refer to [32] for more information. SOM will help in understanding the distribution structure of the solutions in the population. The solutions which are closer in the input space, come closer to each other in the output space (neuron grid in SOM).

### Genetic operators

In any evolutionary algorithm, genetic operators help in generating new solutions. This set of new solutions forms a new population, *P*′. In our framework, from each solution, a new solution is generated using three genetic operators: mating pool generation, mutation, and, crossover. These genetic operators are described below:

#### Mating pool generation

Using this operator, mating pool is constructed for each solution. It consists of a set of solutions which can mate to generate new solutions. For its constructions, neighboring solutions are identified using the trained SOM. Let us assume that we want to generate a new solution for current solution denoted as $\vec{x_{current}}$. Let *β* be some threshold probability. Then its construction steps are described below:

1. Identify the winning neuron ‘*h*′ in the SOM grid for $\vec{x_{current}}$ using the shortest Euclidean distance criterion as $b=\text{arg}\;min_{1\leq u\leq U}\|\vec{x_{current}}-{\vec{w}}^{u}\|$, where ${\vec{w}}^{u}$ is the weight vector of *u*^*th*^ neuron, *U* is the total number of neurons.
2. The solutions mapping to the neighboring neurons are identified by calculating the Euclidean distances between the position vector of neuron ‘*h*′ and other neurons’ position vectors.
3. A random probability, *r*, is generated.
4. If *r* < *β*, then indices of the neurons are sorted based on minimum distance to winning neuron (h). Then, fix number of solutions mapped to sorted neuron indices are extracted to form a mating pool.
5. If *r* > *β*, then all solutions in the population are considered as a part of the mating pool.

Note that *r* < *β* and *r* > *β* exhibit the exploitation and exploration behaviour of the evolutionary algorithm, respectively. These are necessary phenomenon to explore the search space efficiently.

#### Mutation and crossover

Mutation and crossover operations result in the change in the values of the components of the current solution ${\vec{x}}_{current}$, thus, generating new solution corresponding to ${\vec{x}}_{current}$. But, before performing the operation, three random solutions, ${\vec{x}}_{r1}$, ${\vec{x}}_{r2}$ and ${\vec{x}}_{r3}$, from its constructed mating pool are selected and then a probability prototype vector is generated. If the component value of the prototype vector is found to be higher than some random probability lying between 0 to 1, then, that component value is replaced by 1 and vice versa. For more information, reader can refer to the paper [16].

It is important to note that during generation of the new solution, *y*″, all possible combinations of the mating pool (randomly chosen solutions ${\vec{x}}_{r1}$, ${\vec{x}}_{r2}$, ${\vec{x}}_{r3}$) are tried, and mutation and crossover are performed against each combination and then constraint of summary length is checked. It may be possible that more than one combination may satisfy the constraint. In that case, only that combination is selected which is close to length constraint (considering the maximum number of words in the summary).

### Selection of the best |*P*| solutions for next generation

**Fig 2:**
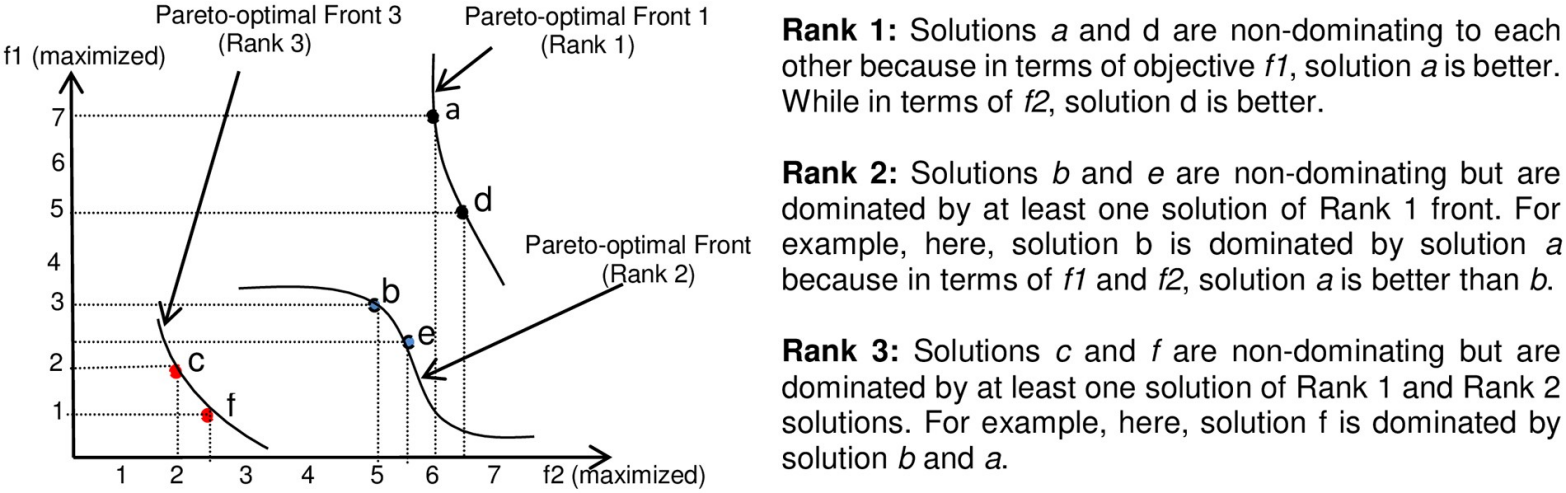
Figure showing dominance and non-dominance relationships in two objective space. Here, both the functions have to be maximized.

This step includes selection of the best |*P*| solutions out of the old population (*P*) and new population (*P*′). Note that size of population *P*′ is equal to population P. To perform this operation, non-dominated sorting (NDS) and crowding distance operator (CDO) of NSGA-II are utilized [14]. NDS includes assignment of ranks to different solutions based on their objective functional values and puts them in different fronts. This phenomenon is shown in Fig 2. CDO identifies the solutions in a front which reside in more crowded region using the nearby solutions in objective space. Best solutions are selected based on their rankings until a desired number of solutions are obtained. In case of a tie in a front, solution having the highest crowding distance is given priority.

Example: Consider a problem where two objectives/fitness functions are to be maximized. Let the size of the population P be 3, i.e., |*P*| = 3 and the objective functional values are (6, 7), (5, 3), (2, 2) corresponding to solutions *a*, *b* and *c*, respectively. Let *d*, *e*, and *f* be the new solutions generated after applications of genetic operators. Let their fitness functional values be (6.5, 5), (5.5, 2.3), and (2.5, 1), respectively. After merging, total number of solutions will be 6 out of which only 3 solutions are passed to the next generation. Firstly, these solutions are ranked using NDS. After calculating ranking as per NDS algorithm, rank-1 solutions are {*a*, *d*}; rank-2 solutions are {*b*, *e*} and rank-3 solutions are {*c*, *f*}. As rank-1 includes two solutions, therefore they will be passed for next generation. Now, only (3 − 2) = 1 solution is left to be chosen from rank-2 set of solutions. Therefore, to select (3 − 2) = 1 solution, crowding distance operator is applied to rank-2 solutions and thus (3 − 2) = 1 solution is selected having highest crowding distance.

### Updation of SOM training data

In this step, training data for SOM is updated. In the next generation, SOM will be trained using those selected solutions (out of the best solutions selected in previous step) which have not been seen before. It is important to note that updated weight vectors of the neurons in the current generation will now be treated as the initial weight vectors of the neurons in the next generation.

### Termination condition

For any iterative procedure, termination condition is required. Therefore, in our work, proposed algorithm is repeated until a maximum number of generations (iterations), *g*_*max*_ is reached. This step is shown by diamond box in Fig 1.

### Selection of single best solution and generation of summary

At the end of the final generation, a set of non-dominated solutions on the final Pareto optimal front are generated by our MOO-based algorithm. Here, Pareto optimal front means all solutions are having equal importance to each other. Thus, it provides a flexibility to a decision maker to select a solution based on his/her requirement. In this paper, firstly, we have generated summaries corresponding to different solutions and then selected that solution which has the highest ROUGE-2 score. This is done to illustrate that our proposed approach can produce the best solution having highest Rouge score in comparison to the state-of-the-art techniques. We have reported the average Rouge score values corresponding to the best solutions (selected based on the highest ROUGE-2 values) for all documents. It also helps in proper comparison with existing methods which produce only a single solution.

Note that to calculate the Rouge score, gold/reference summary is used, which may not be available in real time situations. Therefore, a single solution from the final Pareto front should be selected after considering other criteria which do not use any supervised information. To address this issue, we have explored various methods to select the best solution. Let us name the approaches making use of supervised (available gold summary information) and unsupervised information for selection of single best solution from the final Pareto optimal front as SMaxRouge and UMaxRouge, respectively. The methods explored under UMaxRouge policy are explained below:

1. Maximum values of six different objectives functions and their combinations: coverage (MaxCov), readability (MaxRead), sentence length (MaxSenLen), sentence position (MaxSenPos), similarity with title (MaxSimTitle), cohesion (MaxCoh). To calculate these, firstly, for all the solutions of the final generation, the single objective function (for example, readability score) is analyzed, and then, the solution having the highest value based on chosen single objective function is considered as the best solution. Some combinations of these objective functions are also explored. In this case also, the solution with the highest value is considered as the best solution. For example:
  - MaxWeightSumAllObj: In this approach, summation of all objective functional values optimized in our approach is considered.
  - MaxWeightSum2Obj: In MaxWeightSum2Obj, the summation of two objective functions, namely, sentence position and sentence similarity with the title is considered.
  - MaxWeightSum3Obj: This is similar to MaxWeightSum2Obj. Only difference is that we have added one more objective function namely, cohesion.
2. Ensemble approach (EnSem): In this approach, we have firstly considered all the sentences which are present in the summaries corresponding to all generated rank-1 solutions of the final Pareto optimal front. Then the frequency of occurrence of each of these sentences over different summaries corresponding to different rank-1 solutions is calculated as per Eq 15. Sentences are then sorted based on their frequencies of occurrence and those are added one by one as per their sorted order in the final summary until the desired length is reached.
  Let |*PS*| is the number of rank-1 solutions, *PSS* is the set of all unique sentences present in the summaries corresponding to *PS* number of solutions. Let us assume that we want to count the frequency of occurrence of *ith* sentence, i.e., *sent*_*i*_, belonging to *PSS*. Then, the following equation is followed:
  $$\begin{array}{c} count_{sent_{i}}=\sum_{k=1}^{\left|PS\right|}B\;and\;B=\{\begin{array}{cc} 1, & \text{if}\;sent_{i}\in PS_{k}\\ 0, & \text{otherwise} \end{array} \end{array}$$ (15)
  where, *PS*_*k*_ is the *kth* summary corresponding to *kth* solution of a document. Same equation (the above Eq) was followed to calculate the count of remaining sentences belonging to *PSS*.
  Two other variations of the ensemble approach are also tried. After collecting the sentences of rank-1 solutions (merged pool), they are sorted based on (a) maximum length; (b) maximum sentence to title similarity. For both cases (a) and (b), final summary is generated by adding the sentences from the merged pool one by one following their sorted order until the desired length is reached. In this paper, the approaches corresponding to (a) and (b) are named as *EnSemMaxLen* and *EnSemMaxSentTitle*, respectively.
3. Sentence and Word embedding (MinReconsError): This approach is based on the semantic similarity between the document and the generated summary. The motivation behind this idea is to check whether the generated summary can represent the central theme of the document or not. The solution having maximum semantic similarity [53, 64] will be considered as the best solution. In this approach, firstly, we generate the sentence vectors of all sentences present in a particular document by averaging the word vectors (word-embedding) of the words present in the sentences. To get the word vectors, we have used the pre-trained word2vec model on [53] *GoogleNews* corpus which contains 3 billions words and each word vector is of 300 dimension. A document theme is represented by averaging the sentence vectors of that document. Then the similarity is calculated between sentences present in the summary and document theme vector. The solution with summary having highest similarity will be treated as the best solution. In other words, we can say that a solution will be treated as the best solution if it has the minimum reconstruction error (ReconsError) which is defined as
  $$\begin{array}{c} ReconsError_{j}=\sum_{i=1}^{K}\|DocVec-SentVec_{i}{\|}_{2} \end{array}$$ (16)
  where *DocVec* is the vector representing document’s theme, *SentVec*_*i*_ is the *ith* sentence vector of *jth* summary (or summary corresponding to *jth* solution), *K* is the number of sentences in *jth* summary, ∥*DocVec* − *SentVec*_*i*_∥_2_ is the Euclidean distance between document vector and *ith* sentence vector. In the current paper, we name this approach as MinReconsErrorWord2vec.
  Performing the averaging of word vectors to get the sentence vector and then averaging the sentence vectors to obtain the document vector, somehow reduce the semantics of sentence and document [65] vectors. Therefore, we have tried another approach based on Doc2vec [66]. Its performance is shown to be good when trained on large corpora with pre-trained word-embedding [66]. From the trained model, we can directly get the document vector and sentence vector [67]. Here also we want to minimize the reconstruction error between document vector and generated summary as mentioned in Eq 16. Let us name this approach as MinReconsErrorDoc2vec.
4. Maximum distance from the origin (MaxObjDistOrigin): As six objective functions used in our proposed approach are of maximization type; therefore, here, we have calculated the Euclidean distance between the origin having position (0, 0, 0, 0, 0, 0) and objective functional values of the solution. The solution having the largest distance is selected as the best solution.

Note that, the sentences, present in the final summary, are reported based on their occurrences in the original document. For example, the sentence which appears first in the document will be the first sentence in the summary.

## Experimental setup

This section presents the datasets used for the experimentation, evaluation metrics to measure the performance, comparing methods, followed by parameter settings. All the proposed approaches were implemented on Ubuntu server having Intel Xeon CPU 2.20 GHz with 256 GB of RAM.

### Datasets

In order to show the effectiveness of the proposed approach and to show that performance not only depends on the chosen objective functions, but, also depends on the type of similarity/dissimilarity measures used, two benchmarks datasets namely, DUC2001 and DUC2002 from Document Understanding Conference (https://www-nlpir.nist.gov/projects/duc/data.html0) are used. These contain 309 and 567 news reports (in the form of documents), respectively, written in English. For each document, the original/actual summary is available in approximate 100 words for single document summarization. In addition to these datasets, we have also used the CNN dataset [8, 23] which contains news articles collected from CNN news site https://edition.cnn.com/. It consists of 3000 news articles/documents out of which only 50 articles are made available on https://sites.google.com/view/doceng19-extesu/home?authuser=0 by the authors (at the time of submission). Their actual summary includes 3-4 sentences on an average. Note that our proposed algorithm is fully unsupervised in nature in the sense that it does not use any actual summary information for generating the summary. Actual summary is utilized only for evaluation of our generated summary at the end of execution of our algorithm. A brief description of the used datasets is provided in Table 1.

**Table 1:** Brief descriptions of datasets used. Here, #*DocSentences* is the total number of sentences in the document.

	DUC2001	DUC2002	CNN
#Topics	30	59	11
#Documents	309	567	50
Source	TREC	TREC	CNN
length of summary	100 words	100 words	10% × *DocSentences*

### Comparing methods

We have compared our proposed system with 13 existing systems. Some methods use supervised approaches, while, others used neural network. Some of the comparing algorithms are also based on optimization techniques to improve the ROUGE score. The names of the existing systems used for comparison are Unified Rank [68], MA-SingleDocSum [9], Manifold Ranking [22], QCS [25], CRF [10], NetSum [11], SVM [20], DE [6], FEOM [26], SummaRuNNer [13], NN-SE [12], COSUM [28], ESDS-GHS-GLO [27]. These works except [12, 13] make use of both DUC2001 and DUC2002 datasets for reporting the performance of summarization systems. In addition to these methods, in paper [23], five regression-based methods are proposed, namely, LeastMedSq, Linear Regression, MLP Regressor, RBF Regressor, and SMOreg, which differ in terms of machine learning classifier used. Out of these regression-based models, Linear Regression and LeastMedSq performed the best for DUC2001 and DUC2001 datasets, respectively. Therefore, these best methods are also considered for comparison purpose. Note that [12, 13] make use of only DUC2002 dataset. Therefore, for a fair comparison, results are directly taken from these reference papers. Above discussed techniques are already described in literature survey.

### Evaluation metrics

To evaluate the performance of the proposed architecture, we have utilized the ROUGE measure [69]. It measures the overlapping units between the actual/gold summary and our predicted summary. More will be the ROUGE score, closer will be our summary with respect to the actual summary. The mathematical definition of ROUGE score is defined below:

$$\begin{array}{c} ROUGE-N=\frac{\sum_{S\in Summary_{actual}}\sum_{N-gram\in S}Count_{match}\left(N-gram\right)}{\sum_{S\in Summary_{actual}}\sum_{N-gram\in S}Count\left(N-gram\right)} \end{array}$$ (17)

Where N represents the length of n-gram, *Count*_*match*_(N-gram) is the maximum number of overlapping N-grams between actual summary and the generated summary, *Count*(N-gram) is the total number of N-grams present in the actual summary. In our experiment, N takes the values of 1 and 2 for ROUGE−1 and ROUGE−2, respectively.

In addition to the ROUGE score, we have also reported another evaluation measure namely, BLEU or the Bilingual Evaluation Understudy [70]. It is generally used in machine translation system and compares a candidate translation of text to one or more reference translations, but, can also be used in text summarization task. It also counts the n-gram-overlap between system generated summary and available actual summary, but, with one difference: ROUGE-N considers N-grams (1-gram or 2-gram etc.) at a time, while, BLUE considers various sizes of N-grams (in our case, 1-gram, 2-gram, 3-gram, 4-gram) simultaneously to compute the same. For mathematical definition of BLUE, the reader can refer to [70]. It is important to note that the existing work doesn’t report BLUE score, therefore, we have reported the same only for our best proposed approaches when applied on three datasets.

### Parameter settings

Different parameter values used in our proposed framework are- *DE parameters*: ∣*P*∣ = 40, mating pool size = 4, threshold probability in mating pool construction (*β*) = 0.7, maximum number of generations (*g*_*max*_) = 25, crossover probability (CR) = 0.2, b = 6, F = 0.8. *SOM parameters*: initial neighborhood size (*σ*_0_) = 2, initial learning rate (*σ*_0_) = 0.6, training iteration in SOM = ∣P∣, topology = rectangular 2D grid; grid size = 5 × 8. Sensitivity analysis on DE parameters and SOM parameters can be found in [16] and [32], respectively. Inspired by these works, similar values of parameters are utilized in the current work. Importance factors/weight values assigned to different objective functions: *α* = 0.25, *β* = 0.25, *γ* = 0.10, *δ* = 0.11, λ = 0.19, *ϕ* = 0.10; *System summary*: length (in words) = 100 words. In most of the existing literature [16, 62], similar weight values of importance factors are considered. Results obtained are averaged over 10 runs of the algorithm. Word Mover Distance makes use of pre-trained word2vec model on *GoogleNews* (https://github.com/mmihaltz/word2vec-GoogleNews-vectors) corpus to calculate the distance between two sentences.

## Results and discussion

Table 2 reports the ROUGE scores obtained by our proposed approaches using different similarity/dissimilarity measures (NGD, CS, WMD) and different state-of-the-art methods on DUC2001 and DUC2002 data sets. Note that these results are generated by our proposed approach with SMaxRouge strategy of selection of a single best solution from the final Pareto optimal front as mentioned in section ‘Selection of Single Best Solution and Generation of Summary’. To illustrate the utility of incorporating SOM based genetic operators in the DE process, results are also reported for multi-objective binary DE-based summarization approach with standard genetic operators of DE (without using SOM). It can be observed that our approaches using discussed similarity/dissimilarity measures outperforms all other approaches for both the data sets in terms of ROUGE-1 and ROUGE-2 scores. The best ROUGE scores as reported in Table 2 for both the datasets were obtained using Approach-1 with SOM-based genetic operators and WMD as the similarity measure. Thus it can be concluded from obtained results that the use of different sentence similarity/dissimilarity measures and self-organized multi-objective differential evolution for optimization indeed helps in achieving improved performance.

**Table 2:** ROUGE scores attained by different methods for DUC2001 and DUC2002 data sets. Here our proposed methods are executed using Normalized Google Distance (NGD), Cosine Similarity (CS) and Word Mover Distance (WMD), and, SMaxRouge strategy is used for selecting a single best solution from the final Pareto front. Here, † denotes the best results; it also indicates that results are statistically significant at 5% significance level; xx indicates results are not available in reference paper. For LeastMedSq and Linear Regression methods, results in the reference paper are presented up to 4 decimal points, therefore, to make a fair comparison up to 5 decimal points, we have added 0 as the last decimal digit such that their results remain unchanged. Similar case also applicable to NN-SE and SummaRuNNer methods.

		DUC2001		DUC2002	
		ROUGE-2	ROUGE-1	ROUGE-2	ROUGE-1
Approach-1 (NGD)	With SOM	**0.26949**	**0.47699**	**0.27846**	**0.50225**
	Without SOM	**0.26742**	**0.47521**	**0.27705**	**0.50191**
Approach-2 (NGD)	With SOM	**0.26774**	**0.47291**	**0.27519**	**0.49899**
	Without SOM	**0.26265**	**0.46762**	**0.27654**	**0.50162**
Approach-1 (CS)	With SOM	**0.26459**	**0.47554**	**0.27649**	**0.50624**
	Without SOM	**0.25282**	**0.46289**	**0.27292**	**0.50050**
Approach-2 (CS)	With SOM	**0.26209**	**0.47398**	**0.25961**	**0.49159**
	Without SOM	**0.26629**	**0.47862**	**0.27319**	**0.50147**
Approach-1 (WMD)	With SOM	***0.29238*^†^**	***0.50236*^†^**	***0.28846*^†^**	***0.51662*^†^**
	Without SOM	**0.28930**	**0.49486**	**0.28556**	**0.51441**
Approach-2 (WMD)	With SOM	**0.28462**	**0.49863**	**0.28520**	**0.51538**
	Without SOM	**0.28190**	**0.48877**	**0.28656**	**0.51406**
COSUM [28]	-	0.20123	0.47274	0.23092	0.49083
ESDS-GHS-GLO [27]	-	0.19574	0.45403	0.22142	0.47903
MA-SingleDocSum [9]	-	0.20142	0.44862	0.22840	0.48280
DE [6]	-	0.18523	0.47856	0.12368	0.46694
UnifiedRank [68]	-	0.17646	0.45377	0.21462	0.48487
FEOM [26]	-	0.18549	0.47728	0.12490	0.46575
NetSum [11]	-	0.17697	0.46427	0.11167	0.44963
CRF [10]	-	0.17327	0.45512	0.10924	0.44006
QSC [25]	-	0.18523	0.44852	0.18766	0.44865
SVM [20]	-	0.17018	0.44628	0.10867	0.43235
Manifold Ranking [22]	-	0.16635	0.43359	0.10677	0.42325
Linear Regression [23]	-	0.21104	0.46374	0.23924	0.49784
LeastMedSq [23]	-	0.20794	0.46204	0.23964	0.49824
NN-SE [12]	-	xx	xx	0.23200	0.47400
SummaRuNNer [13]	-	xx	xx	0.23900	0.45400

As Approach-1 (as per results of Table 2), utilizing word mover distance, performs best, therefore, we have evaluated the same approach on the third dataset namely, CNN. The corresponding results are reported in the Table 3. Here, results are shown only for 50 articles. Although, there exist papers [8] and [23] which use 400 and 3000 articles of CNN, respectively, but, it will be unfair to compare our results with these papers due to unavailability of complete dataset for CNN. Moreover, the codes of these papers, [8] and [23], are not available. Therefore, for comparison purpose, we have used our own Approach-2 utilizing WMD. Note that results of Approach-1 and Approach-2 for CNN dataset are shown using with and without SOM-based genetic operators. From Table 3, it can be observed that Approach-1 using WMD as a dissimilarity measure and SOM as a genetic operator, performs the best which was also the case for DUC2001 and DUC2002 datasets.

**Table 3:** ROUGE scores attained by proposed Approach-1 and Approach-2 utilizing word mover distance (WMD) on CNN dataset. Here, SMaxRouge strategy is used for selecting a single best solution from the final Pareto front.

		ROUGE-2	ROUGE-1
Approach-1 (WMD)	With SOM	**0.67431**	**0.72478**
	Without SOM	0.42772	0.45117
Approach-2 (WMD)	With SOM	0.66146	0.71166
	Without SOM	0.46144	0.48357

### Comparison of results using BLEU score

The results in terms of BLEU score corresponding to three datasets are reported in Table 4. Note that from the final set of Pareto optimal solutions, a particular solution *sol1*, maybe best w.r.t. ROUGE-2 F1-measure, but, may not be best w.r.t. BLUE score. Therefore, the average BLUE score reported in the Table is obtained by selecting the best solution from the final set of Pareto optimal solutions based on maximum BLUE score. As the existing approaches do not report the BLUE score values; therefore, for the purpose of comparison, we have considered our best approach, Approach-1 utilizing WMD in comparison with Approach-2 (WMD). Here also, results of these approaches are illustrated using SOM and without SOM-based genetic operators. From this Table, it can be observed that Approach-1 (WMD) using SOM-based genetic operators performs best and is able to attain BLUE score values of 0.32623, 0.21641, and, 0.62009 for DUC2001, DUC2001 and CNN datasets, respectively.

**Table 4:** BLUE scores attained by proposed Approach-1 and Approach-2 utilizing word mover distance (WMD) on three datasets. Here, SMaxRouge strategy is used for selecting a single best solution (based on maximum BLEU score) from the final Pareto front.

		DUC2001	DUC2002	CNN
Approach-1 (WMD)	With SOM	**0.32623**	**0.21641**	**0.62009**
	Without SOM	0.23635	0.20038	0.51075
Approach-2 (WMD)	With SOM	0.26913	0.20357	0.620061
	Without SOM	0.24217	0.20388	0.48712

**Fig 3:**
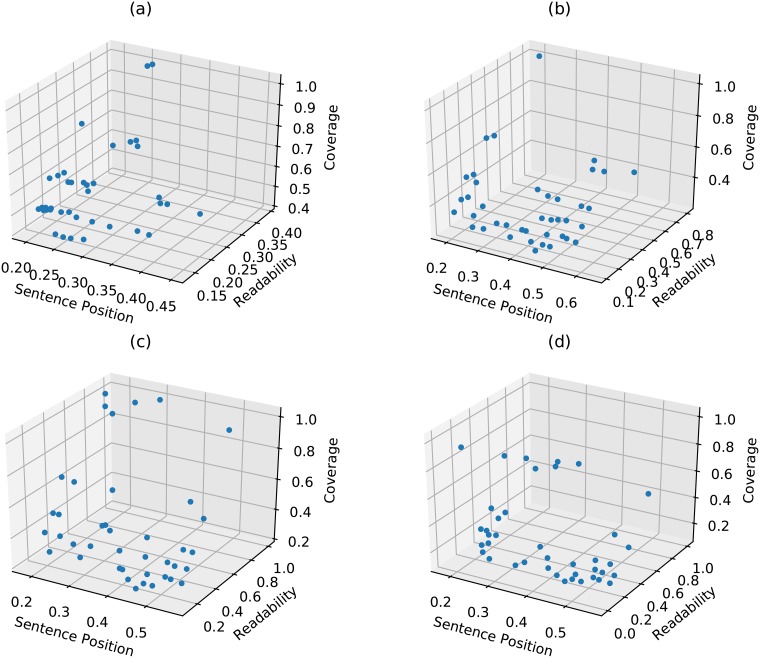
Pareto optimal fronts obtained after application of the proposed approach. Here, Proposed approach refers to Approach-1 (WMD) with SOM-based operators. Sub-figures (a), (b), (c) and (d) are the Pareto optimal fronts obtained after first, fourteen, nineteen and twenty-fifth generation, respectively. Red color dots represent Pareto optimal solutions; three axes represent three objective functional values, namely, sentence position, readability, coverage.

As any evolutionary algorithm generates Pareto optimal solutions in the final generation, therefore, we have shown the Pareto optimal fronts obtained (over one random document of DUC2001/DUC2002) after the application of the proposed *Approach-1 (WMD) with SOM-based operators* in the Fig 3. These fronts correspond to first, fourteen, nineteen and twenty-fifth generations. Note that it is difficult to plot Pareto optimal fronts for six objective functions. Therefore, we have shown the projected Pareto optimal fronts in three objective space (as shown in Fig 3). The following three subsections will discuss the results obtained using different distance/similarity measures on DUC2001 and DUC2002 datasets.

#### Discussion of results obtained using normalized google distance (NGD)

In Table 2, considering all cases (both approaches, with SOM and without SOM based genetic operators), our results beat other existing methods. The best ROUGE scores for both the datasets were obtained using Approach-1 with SOM-based genetic operators. On comparing the results of Approach-2 with SOM and without SOM-based operators for DUC2002 dataset, it was observed that ROUGE-2 and ROUGE-1 scores are higher in case of Approach-2 without SOM-based operators. But, the difference is not much significant when compared using *SOM* based operators.

### Discussion of results obtained using cosine similarity (CS)

In Table 2, considering all cases (both approaches, ‘with SOM’ and ‘without SOM’ based genetic operators), it can be concluded that our proposed approaches outperform other existing methods. Out of both operators in Approach-1 utilizing WMD, ‘with SOM’ operator perform well. On comparing the results of Approach-2 using ‘with SOM’ and ‘without SOM’ based operators for the DUC2002 dataset, it was observed that ROUGE-2 and ROUGE-1 scores are higher in case of ‘without SOM’ based operators. However, the difference is not much significant when compared using *SOM* based operators.

### Discussion of results obtained using word mover distance (WMD)

In Table 2, considering all cases (both approaches), it was found that Approach-1 obtains the best ROUGE scores with SOM-based genetic operators for both the datasets. This result is also the best when comparing with other similarity/dissimilarity measures. One of the reasons behind this improved performance is the ability of WMD in capturing semantic relationships between sentences. Another possible reason is the use of SOM-based operators which helps the algorithm to reach the optimal solution having good ROUGE scores. Time taken to generate summary using Approach-1 with SOM-based operators for DUC2001 is 32 second/document, while the same approach without SOM based operators takes 29 second/document. For DUC2002, Approach-1 with SOM and without SOM-based operators, take the almost same time, i.e., 20 second/document. Note that these reported times exclude the time taken to calculate similarity/ dissimilarity between two sentences, which is approx 10-20 second in case of WMD.

#### Analysis on conflicting behaviours of the two objective functions

It should be noted that ROUGE score measures the informativeness of the summary obtained and makes use of the actual summary, thus, can be considered as a type of extrinsic measure. But, to measure the quality of the summary, we have also reported an intrinsic measure (independent of actual summary), readability, of the summary which was one of objective functions in our proposed approach. This was done because it is one of the major concerns in any summarization system. The corresponding results for DUC2001, DUC2001, and CNN datasets, are shown in Table 5. These results correspond to the summaries obtained whose ROUGE scores are reported in Tables 2 and 3. Here also, we have used our best approach (Approach-1) utilizing WMD as a dissimilarity measure. For comparison, we have used the Approach-2. Results are shown using SOM and without SOM-based genetic operators. Higher the readability score, easier will be the understanding of the summary for the end-users or in other words, more readable it is. From Table 5, it can be inferred that maximum readability scores of 0.43362 and 0.44392 were obtained by Approach-1 (WMD) using SOM-based operator for DUC2001 and DUC2002 dataset, respectively. On the other hand, for CNN dataset, maximum readability score was attained by the same approach, but, without using SOM-based genetic operator. As in any multi-objective optimization based approach, objective functions are generally conflicting in behaviour or in other words, one solution may be good in terms of one objective, while, may not be good w.r.t. another objective function. We can also say that increase in one, may decrease in another objective functional value in a single solution. Therefore, we have reported another intrinsic measure, i.e., *coverage* (COV), in the same Table. COV is another concern of any summarization system and is used as one of the objective functions in our optimization strategy. After observing the results, it can be inferred that Approach-1 (WMD) utilizing SOM has highest coverage of 0.97735 for CNN dataset, but, for remaining datasets, highest coverage was obtained using Approach-1 (WMD) using without SOM-based operators. These results illustrate the conflicting behaviour of coverage and readability. Note that we have omitted the discussion on other objective functions to avoid a longer discussion.

**Table 5:** Average readability and coverage scores of the summaries obtained by our proposed approaches utilizing WMD on three datasets. Here, the used summaries are obtained using SMaxRouge strategy.

		DUC2001		DUC2002		CNN	
		READ	COV	READ	COV	READ	COV
Approach-1 (WMD)	With SOM	**0.4336**	0.40735	**0.44392**	0.39236	0.35441	**0.97735**
	Without SOM	0.42982	**0.65082**	0.43363	**0.69223**	**0.39277**	0.62694
Approach-2 (WMD)	With SOM	0.40392	0.41195	0.39864	0.39374	0.35274	0.97183
	Without SOM	0.41313	0.40057	0.30525	0.32416	0.37567	0.60468

### Study on different methods of selecting a single best solution from final pareto front

In Table 2, we have shown the best results produced by our proposed approaches utilizing SMaxRouge strategy for selecting a single best solution from the final Pareto front. But, in real time situations, actual summary may not be available. Therefore, we have explored various unsupervised methods under UMaxRouge strategy to generate a single summary out of multiple solutions on the final Pareto optimal front as discussed in section ‘Selection of Single Best Solution and Generation of Summary’. Corresponding results are reported in Table 6. It is important to note that among-st different proposed approaches, Approach-1 (WMD) performs the best with SMaxRouge strategy for the selection of single best solution; therefore, unsupervised methods are explored under this approach only. It can be observed from Table 6 that the method, MaxWeightSum2Obj, is able to beat the remaining approaches for DUC2002 dataset; having Rouge-1 and Rouge-2 scores of 0.51191 and 0.24871 (using SOM based operators), respectively, but, these scores are less than Rouge-1 and Rouge-2 scores of 0.51662 and 0.28846, respectively, which were the best results attained by SMaxRouge strategy. For DUC2001 dataset, using MaxWeightSum2Obj, we obtain better results in terms of Rouge-1 score having value 0.20839, but, it is just close to the best result of existing approaches. But, most of the approaches under UMaxRouge strategy are not able to select the best solution as selected by SMaxRouge strategy. Hence performances of these approaches are poorer compared to SMaxRouge strategy as reported in Table 2.

**Table 6:** ROUGE scores obtained using Approach-1 (WMD) when the best solution is selected using any of the strategies under UMaxRouge strategy. All the strategies explored here for selecting a single best solution from the final Pareto front are unsupervised in nature. Bold entries indicate they are able to beat the state-of-the-art algorithms.

		DUC2001		DUC2002	
		ROUGE-2	ROUGE-1	ROUGE-2	ROUGE-1
MaxCoh	With SOM	0.09268	0.31442	0.11924	0.34899
	Without SOM	0.08949	0.30803	0.16460	0.27372
MaxCov	With SOM	0.13969	0.41237	0.17064	0.45934
	Without SOM	0.16107	0.42382	0.11007	0.24130
MaxRead	With SOM	0.13388	0.38343	0.15633	0.423459
	Without SOM	0.13353	0.38081	0.16276	0.28974
MaxSenLen	With SOM	0.11518	0.37225	0.14217	0.42641
	Without SOM	0.11659	0.37350	0.11830	0.23563
MaxSenPos	With SOM	0.20163	0.43891	0.24859	0.50957
	Without SOM	0.19796	0.43700	0.18503	0.33797
MaxSimTitle	With SOM	0.17096	0.42528	0.20021	0.46747
	Without SOM	0.07824	0.21931	0.16498	0.30265
MaxWeightSumAllObj	With SOM	0.17484	0.42412	0.20669	0.47523
	Without SOM	0.20450	0.45214	0.18319	0.32418
MaxWeightSum2Obj	With SOM	**0.20839**	0.47140	**0.24871**	**0.51191**
	Without SOM	0.20431	0.44477	0.18402	0.33673
MaxWightedSum3Obj	With SOM	0.19723	0.43780	0.24787	0.50997
	Without SOM	0.20518	0.44514	0.33872	0.18752
Ensemble	With SOM	0.12717	0.32238	0.15327	0.37152
	Without SOM	0.12065	0.31312	0.14944	0.36796
EnSemMaxLen	With SOM	0.06512	0.25632	0.09802	0.30849
	Without SOM	0.08931	0.26963	0.09714	0.30733
EnSemMaxSentTitle	With SOM	0.11611	0.30302	0.14499	0.35167
	Without SOM	0.05194	0.22642	0.14267	0.35113
MaxObjDistOrigin	With SOM	0.18474	0.43984	0.21136	0.48370
	Without SOM	0.186083	0.43571	0.162728	0.30669
MinReconsErrorWord2vec	With SOM	0.15695	0.39800	0.19048	0.44749
	Without SOM	0.14409	0.38408	0.18777	0.32736
MinReconsErrorDoc2vec	With SOM	**0.29221**	**0.49990**	**0.28620**	**0.51623**
	Without SOM	**0.28930**	**0.48486**	**0.27142**	**0.50101**

Ensemble based approach in general performs well. But, as there are a large number of non-dominated, a variety of solutions (a solution ‘*sol*_*i*_’ may be good in terms of ‘sentences to title similarity’ objective as compared to ‘*sol*_*j*_’. On the other hand, solution ‘*sol*_*j*_’ may be good in terms of cohesion objective which is of low priority in our approach) and we have considered the sentences belonging to these solutions to generate the final summary, the ensemble approach does not perform better than SMaxRouge strategy.

After observing the results obtained by MaxCoh, MaxCov, MaxRead, MaxSenLen, MaxSenPos, MaxSimTitle approaches of selecting a single best solution (based on maximum value of single objective function) it was concluded that these approaches are also not able to extract the best solution from the final Pareto optimal front. Only the approach, MinReconsErrorDoc2vec is able to perform well and beats the existing algorithms. But, there are slight variations in the results as reported in Table 2. In summary, it can be concluded that solutions selected using MinReconsErrorDoc2vec under UMaxRouge scheme are very similar to those selected by SMaxRouge scheme (refer to Table 2) where available reference/gold summary is utilized for selecting single best solution. Thus performances of the proposed approaches under MinReconsErrorDoc2vec and SMaxRouge strategies are similar. But the MinReconsErrorDoc2vec scheme does not utilize any available supervised information. Thus the use of the MinReconsErrorDoc2vec scheme is recommended with the proposed approaches for selecting the single best solution from the final Pareto front. Note that doc2vec used in this approach was trained using DUC2001, DUC2002, DUC2006 and DUC2007 data sets utilizing implementation available at https://github.com/jhlau/doc2vec under the default parameters mentioned in that link and makes use of pre-trained model on *googlenews* corpus. DUC2006 and DUC2007 are the standard summarization datasets consisting of 50 and 45 document sets, respectively.

### Convergence speed

To demonstrate that our proposed approach converges faster compared to existing algorithms, we have summarized the population size and the number of fitness function evaluation (NFE) used by different algorithms in Table 7. NFE is calculated as:

$$\begin{array}{c} NFE=Population\_size\times Number\_of\_generations \end{array}$$ (18)

The time complexity of any optimization algorithm depends on the number of fitness function evaluations. Table 7 clearly demonstrates that our proposed approach evaluates less or equal number of functions compared to other existing state-of-the-art techniques. Despite of this, the ROUGE score values attained by our proposed approach are better than those attained by the existing techniques. This proves that our approach converges much faster compared to the existing techniques. As our algorithm is based on optimization strategy, therefore, in Table 7, only algorithms based on some optimization strategies are compared.

**Table 7:** Population size and number of fitness evaluation (NFE) used by different optimization approaches. ‘-’ indicates value not mentioned in the reference paper.

	Proposed Approach	COSUM	MA-SingleDocSum	ESDS-GHS-GLO	DE
Population Size	25	200	30	-	200
NFEs	1000	1000	1600	1600	1000

**Fig 4:**
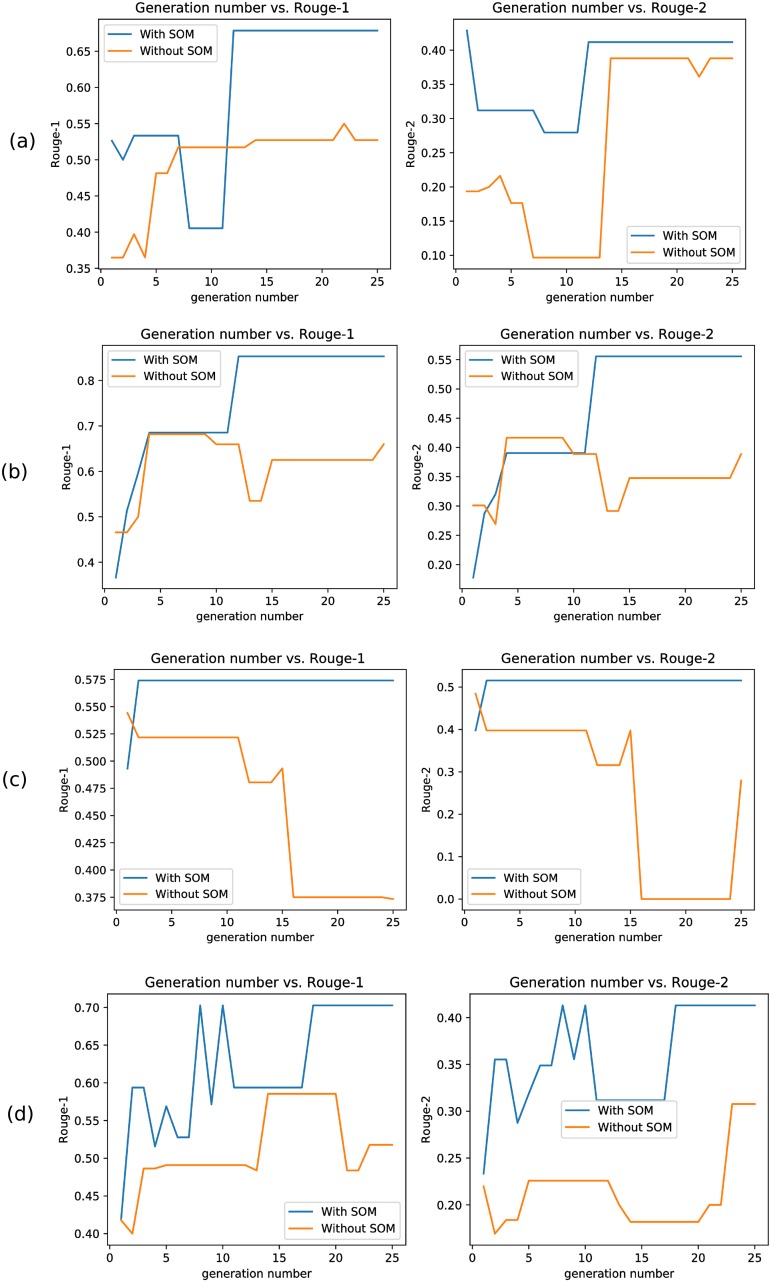
Convergence plots. Sub-figures (a), (b), (c) and (d) show the convergence plots for four random documents. At each generation/iteration, maximum Rouge-1 and Rouge-2 scores are plotted.

We have also shown the convergence plots obtained by our proposed approach for some random documents in Fig 4. Maximum Rouge-1 and Rouge-2 score values attained by our approach over the generations are plotted. These figures show that *Approach-1 (WMD) with SOM* converges to a Rouge-1/Rouge-2 value after a particular iteration (as there is no change in Rouge-1/Rouge-2 score values after that iteration). This also proves the faster convergence of our approach towards the near optimal value of Rouge score (in comparison to other approaches).

### Improvements obtained

We have also calculated the performance improvements obtained (PIO) by our best approach under SMaxRouge strategy to select a single best solution from the final Pareto front in comparison to existing methods using the ROUGE−2 and ROUGE−1 scores and those values are shown in Table 8. These improvements correspond to the best results when using Approach-1 (WMD) with SOM-based operators. Mathematically, PIO is defined as:

$$\begin{array}{c} PIO=\frac{ProposedMethod-OtherMethod}{OtherMethod}\times 100 \end{array}$$ (19)

**Table 8:** Improvements attained by the proposed approach, Approach-1 (WMD) with SOM based operators over other methods considering ROUGE scores. Here, xx indicates non-availability of results on the DUC2001 dataset.

Methods	Improvements obtained by Proposed approach (%)			
	DUC2001		DUC2002	
	ROUGE-2	ROUGE-1	ROUGE-2	ROUGE-1
COSUM	45.30	6.27	24.92	5.25
ESDS-GHS-GLO	49.37	10.64	30.28	7.85
MA-SingleDocSum	45.16	11.98	26.30	7.01
DE	57.85	4.98	133.24	10.64
UnifiedRank	65.69	10.71	34.41	6.55
FEOM	57.63	5.26	130.96	10.92
NetSum	65.21	8.21	158.32	14.90
CRF	68.74	10.38	164.07	17.4
QSC	57.85	12.01	53.72	15.15
SVM	71.81	12.57	165.45	19.49
Manifold Ranking	75.76	15.86	170.18	22.06
Linear Regression	38.57	8.34	20.60	3.78
LeastMedSq	40.63	8.74	20.40	3.70
NN-SE	xx	xx	24.88	8.99
SummaRuNNer	xx	xx	20.70	13.21

Here, improvements obtained by our proposed approach compared to *MA-SingleDocSum* and DE are 45.16% and 4.98%(≡ 5%) respectively, for the DUC2001 dataset, considering ROUGE−2 and ROUGE−1 scores. While for DUC2002 dataset, improvements obtained by our approach compared to *MA-SingleDocSum* and *COSUM* are 26.3% and 5.25%, respectively. After comparing with the latest work on summarization [13] based on neural network, we obtained 20.70% and 8.99%(≡ 9%) improvements over ROUGE-2 and ROUGE-1 scores, respectively, for the DUC2002 dataset. In summary, for DUC2001 dataset, minimum 38.57% and 5.24% improvements are obtained over the existing techniques in terms of ROUGE-2 and ROUGE-1 score, respectively. While for DUC2002 dataset, mimimum 20.60% and 3.70% improvements are obtained over the existing techniques in terms of ROUGE-2 and ROUGE-1 score, respectively.

### Error-analysis

**Fig 5:**
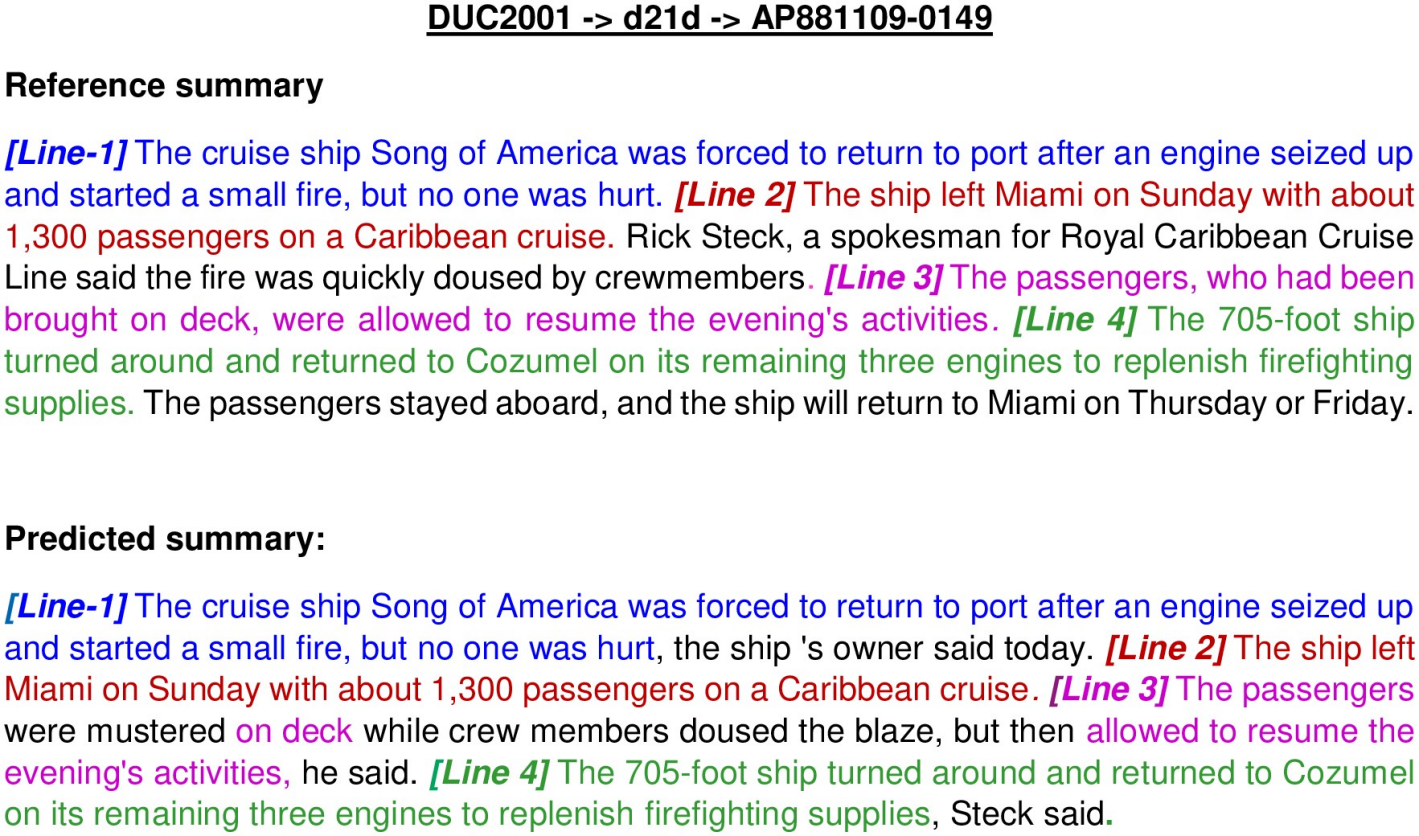
An example of reference summary and predicted summary for document *AP*881109—0149 of topic *d*21*d* under DUC2001 dataset.

In this section, we have thoroughly analyzed the errors made by our proposed approach, Approach-1 (with SMaxRouge strategy of selection of a single best solution from the final Pareto optimal front) with SOM-based operators using WMD as similarity/dissimilarity measure between sentences (as this approach gives the best result). Some random documents from DUC2001/DUC2002 are selected to perform error-analysis from each dataset. It has been observed that proposed approach generates less informative summary if a document length is very large because of length constraint. Some parts of the lines in predicted and reference/actual summary do not match because some sentences in the actual summary were generated by human annotators. In Fig 5, an example of generated summary by our proposed algorithm is shown corresponding to document *AP*881109—0149 of topic *d*21*d* under DUC2001 dataset. The same color shows matching lines, and the beginning of a line is indicated by [*Line*-*number*]. Here, the generated summary covers most of sentences in actual summary having 0.8115 and 0.6383 as ROUGE-1 and ROUGE-2 scores, respectively. Therefore, it is considered as a good summary.

**Fig 6:**
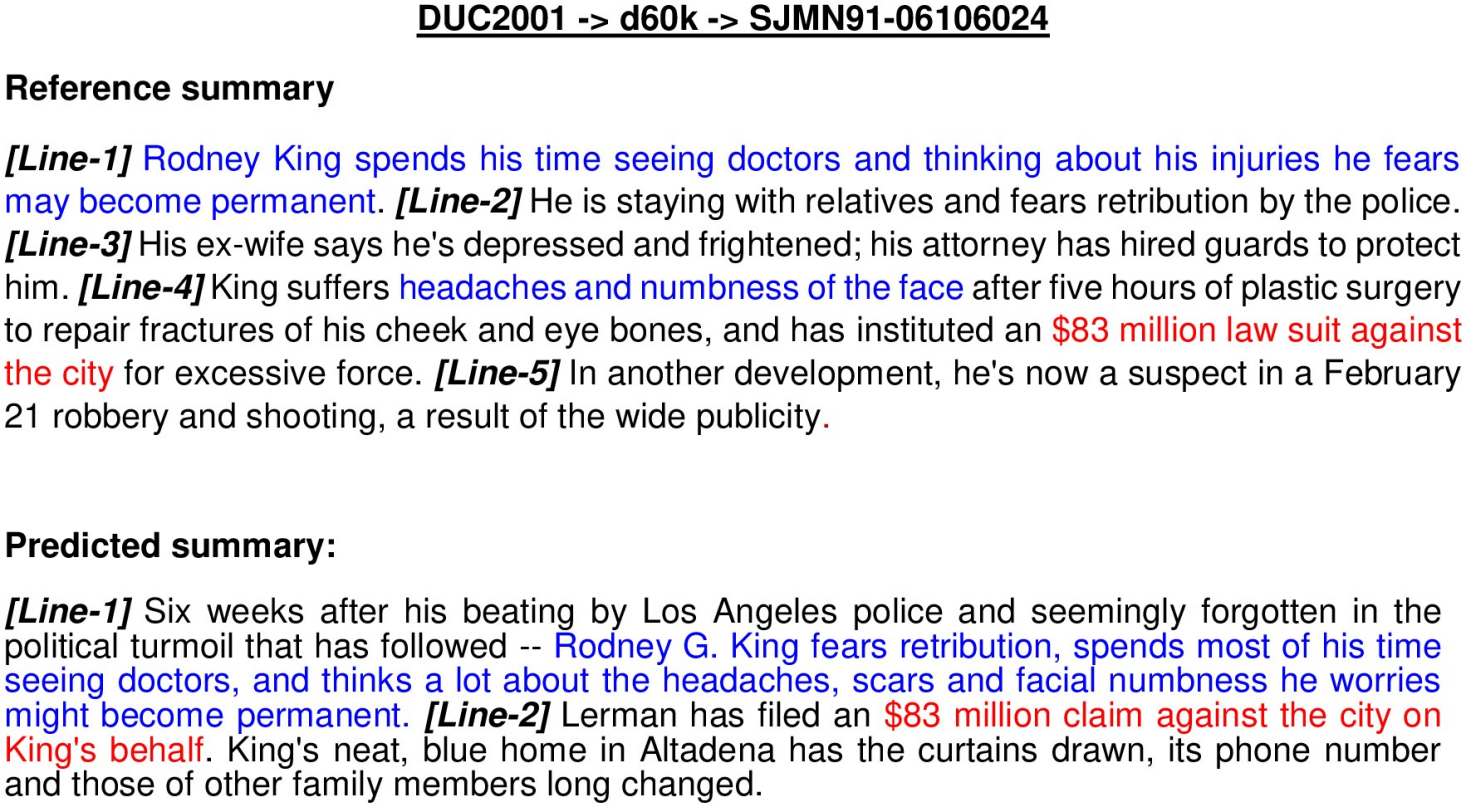
An example of reference summary and predicted summary for document *SJMN*91—06106024 of topic *d*60*k* under DUC2001 dataset.

Fig 6 shows an example of a predicted summary which does not seem to be good, and the corresponding values of ROUGE-1 and ROUGE-2 scores are 0.44 and 0.1276, respectively. The possible reasons could be the generation of reference summary by human annotators. Our developed approach is based on extractive summarization. Therefore, it selects direct sentences from the document to be present in the generated summary, but, it is not capable of restructuring the sentences. For example, consider Line-1 of Fig 6 in the predicted summary which is too long in original document, but is shortened by annotators in *Line* − 1 of reference summary to allow the reference summary to cover other themes of the main document (as more number of words can be added to reach the desired summary length). However, our predicted summary is not able to cover the whole idea of the document as the selection of *Line* − 1 increases the number of the words in summary and not many sentences can be added because of restriction in the number of words in summary.

### Statistical significance t-test

To validate the results obtained by the proposed approach, a statistical significance test named as, Welch’s t-test [71], is conducted at 5% significance level. It is carried out to check whether the best ROUGE scores obtained by Approach-1 (WMD) with SOM-based operators (under SMaxRouge scheme) are statistically significant or occurred by chance. To establish this, we have calculated the p-value using Welch’s t-test among two groups. The first group includes a list of ROUGE-1 (ROUGE-2) values produced by our method after executing it for *Q* (equal to number of comparing methods) times, while, the second group contains a list of ROUGE-1 (ROUGE-2) values by remaining methods. Now, two hypotheses are considered by this t-test namely, the null hypothesis and the alternative hypothesis. The null hypothesis states that there is no significant difference between median ROUGE-1 (ROUGE-2) values of the two groups. On the contrary, alternative hypothesis states that there is significant difference between median ROUGE-1 (ROUGE-2) values of two groups. This t-test provides p-value. Minimum p-value signifies that our results are significant. The p-values obtained are shown in Table 9. Test results support the hypothesis that obtained improvements by the proposed approach are not occurred by chance, i.e., improvements are statistically significant.

**Table 9:** The p-values obtained by Approach-1 (WMD) with SOM based operators (under SMaxRouge scheme) considering ROUGE-1 and ROUGE-2 score values.

Dataset	ROUGE-1	ROUGE-2
DUC2001	0.000152	< 0.00001
DUC2002	0.004183	< 0.00001

### Study on effectiveness of SOM based operators on DUC2001 and DUC2002 datasets

**Fig 7:**
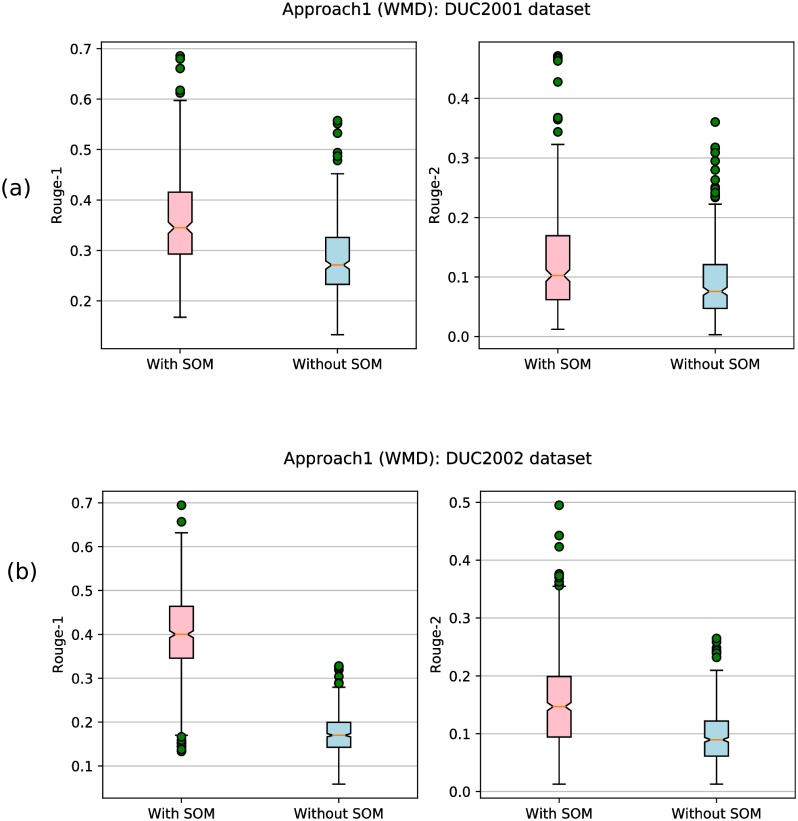
Box plots. Sub-figures (a) and (b) for DUC2001 and DUC2002 dataset, respectively, show the variations of average Rouge-1/Rouge-2 values of highest ranked (rank-1) solutions in each document. In each colored box, the horizontal colored line indicates the median value of rank-1 solutions.

**Fig 8:**
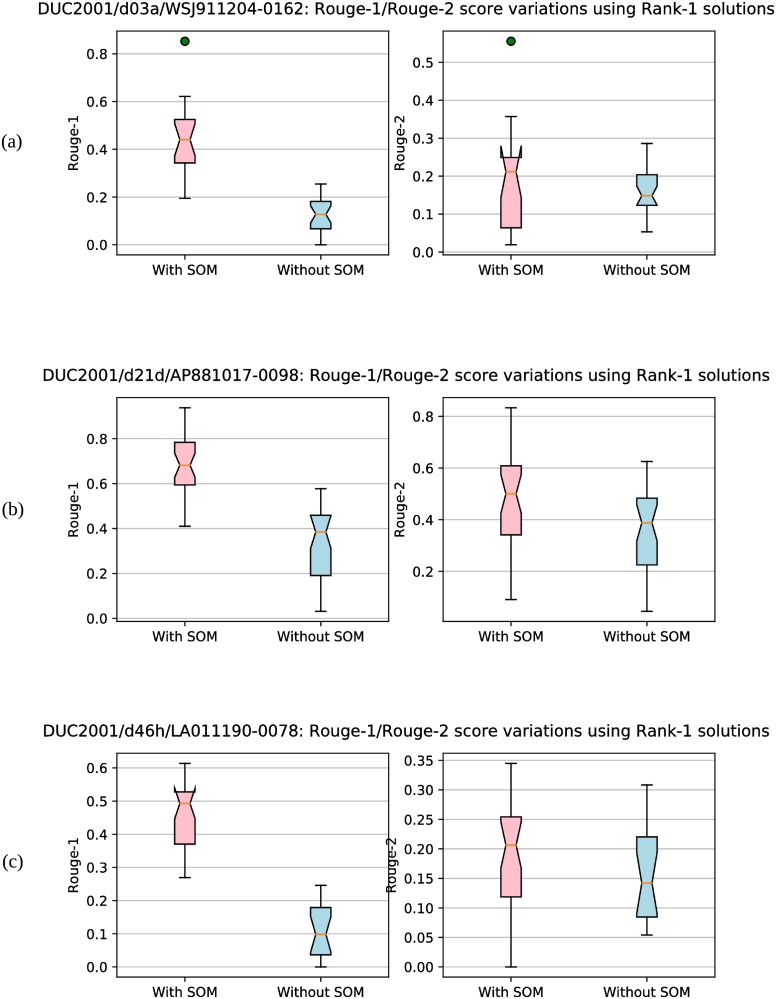
Box plots. Sub-figures (a), (b) and (c) show the Rouge-1/Rouge-2 score variations per document over DUC2001 dataset. In each colored box, the horizontal colored line indicates the median value of Rouge-1/Rouge-2 score using rank-1 solutions of a document.

**Fig 9:**
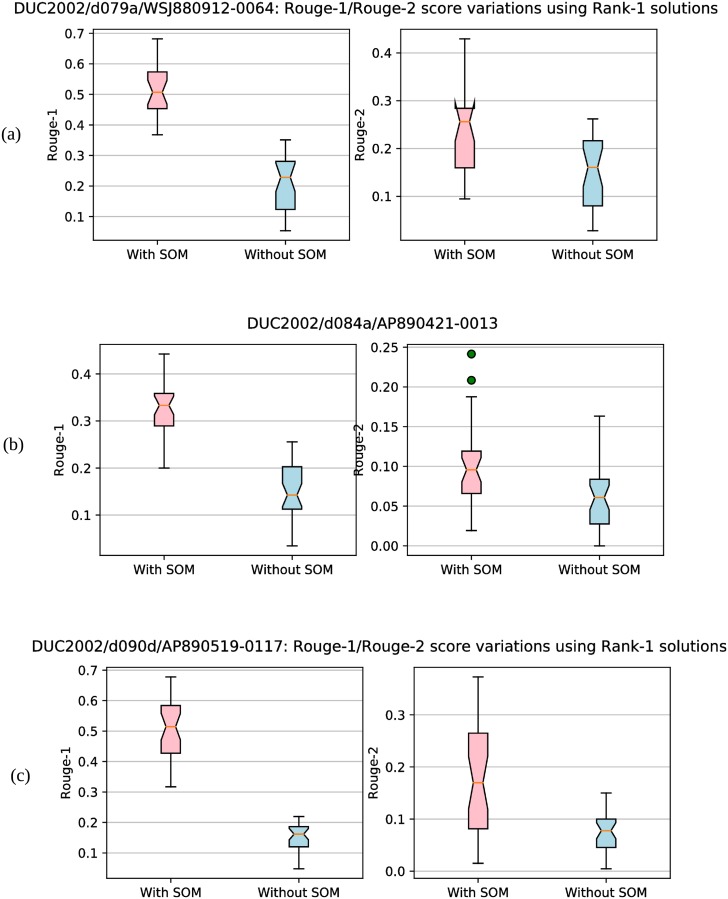
Box plots. Sub-figures (a), (b) and (c) show the Rouge-1/Rouge-2 score variations per document over DUC2002 dataset. In each colored box, the horizontal colored line indicates the median value of Rouge-1/Rouge-2 score using rank-1 solutions of a document.

Note that difference in the Rouge-1/Rouge-2 score values attained by ‘with SOM’ and ‘without SOM’ versions of Approach-1 (WMD) (shown in the Table 2) seems to be very small. In order to further investigate the issue, we have carried out the following analyses: (a) plotted the box plots; (b) performed the t-test. Detailed information about these are given below:

1. Box plots: We have plotted the box plots showing the variations of the average Rouge-1/Rouge-2 values of the highest ranked solutions (Rank-1) produced in the final generation of each document. For example, let *d* be a particular document belonging to DUC2001/DUC2002 dataset and *Q* be the number of rank-1 solutions obtained on the final Pareto optimal front of the final generation in that document, then average Rouge-1 for the document ‘d’ denoted as *Average*_*R*1_*d*_ is calculated as:
  $$\begin{array}{c} Average\_R1_{d}=\sum_{j=1}^{Q}R1_{j} \end{array}$$ (20)
  where, *R*1 and *j* indicate the Rouge-1 score and rank-1 *jth* solution, respectively. Similar steps are followed to calculate the average Rouge-2 value. Following the above process, average Rouge scores are calculated for all the documents. This is done because we have reported the average Rouge-1/Rouge-2 scores of the best solutions of all documents in Table 2 and the best solution is one of the highest ranked solutions. Note that the best results are obtained using Approach-1 (WMD). Therefore, box plots are drawn for this method. From Fig 7(a) and 7(b), it is evident that Approach-1 with SOM-based operators attains better median values of the average of Rouge-1/2 values of rank-1 solutions of all documents for DUC2001 and DUC2002 datasets, respectively, in comparison to those obtained by ‘without SOM based operators’. Also for both the datasets, Approach-1 (WMD) using SOM-based operator covers solutions having a high range of Rouge-1/Rouge-2 values as can be analyzed from the green bullets/points in these figures.
  We have also drawn the box plots for three random documents showing Rouge-1/Rouge-2 variations (with SOM and without SOM based operators) across different rank-1 solutions. These box plots are shown in Figs 8 and 9 for DUC2001 and DUC2002 dataset, respectively. These box plots per document also show the superiority of SOM based operators in covering a high range of Rouge-1 and Rouge-2 score values. At the top of each sub-figure of Figs 8 and 9, super-title is written describing dataset name, topic name and document number under that topic. For example, at the top of Fig 8(a), ‘DUC2001/d03a/WSJ911204-0162’ is written indicating dataset: DUC2001, topic name: d03a and document number: *WSJ*911204—0162.
2. t-test: We have also conducted t-test to show the significant difference between the Rouge recall values obtained by two versions (with SOM and without SOM based operators) of Approach-1 (WMD) under SMaxRouge scheme. The p-values (at 5% significant level) attained by these approaches are reported in Table 10.
  These p-values obtained on DUC2001 dataset clearly show that Approach-1 (WMD), when used with SOM based operator significantly improves the results. But, on DUC2002 dataset, results obtained are not significant as Rouge score values attained by Approach-1 (WMD) *with SOM based operators* are close to those attained by Approach-1 (WMD) *without SOM based operators*. However, from Figs 7–9, it is appropriate to say that there exist a set of documents for which our approach is able to determine good quality solutions with high Rouge scores in less number of iterations when used with SOM based operators.

**Table 10:** The p-values obtained by Approach-1 (WMD) with SOM and without SOM based operators (under SMaxRouge scheme) considering ROUGE-1 and ROUGE-2 score values.

Dataset	ROUGE-1	ROUGE-2
DUC2001	0.024134	0.032038
DUC2002	0.218967	0.238569

### Ranking of methods

We have also calculated the ranking scores of different methods using the Unified Ranking [9] method by considering the individual ranks of different methods with respect to different measures as shown in Table 11. It is created using Table 2. This method was first proposed by Ramiz M. Aliguliyev [72]. While calculating the ranking, we have excluded the rankings of NN-SE, and SummaRuNNer approaches as their results for the DUC2001 dataset are not available in the reference papers. The resultant rank of each method is calculated as follows:

$$\begin{array}{c} Ranking\_score\left(method\right)=\sum_{p=1}^{12}\frac{\left(12-p+1\right)R_{p}}{12} \end{array}$$ (21)

where, the number, 12 denotes the number of methods in comparison including the proposed one, *R*_*p*_ denotes how many times a method appears at the *pth* position. Finally, the method having the highest Ranking_score is assigned the highest rank. From the Table 11, we can see that Approach-1 with SOM based operators, when used with word-mover-distance, is selected at rank 1.

**Table 11:** Ranking of different methods.

Method	*R*_*p*_												Ranking_score	Rank
	1	2	3	4	5	6	7	8	9	10	11	12		
Approach-1 with SOM (WMD)	4	0	0	0	0	0	0	0	0	0	0	0	4.8	1
COSUM	0	2	1	1	0	0	0	0	0	0	0	0	3.4	2
ESDS-GHS-GLO	0	0	0	2	1	0	1	0	0	0	0	0	2.7	3
MA-SingleDocSum	0	1	1	1	0	0	0	0	1	0	0	0	2.8	4
DE	0	1	0	0	0	2	0	1	0	0	0	0	2.5	5
FEOM	0	0	1	0	1	0	2	0	0	0	0	0	2.5	5
UnifiedRank	0	0	1	0	1	0	0	2	0	0	0	0	2.3	6
NetSum	0	0	0	0	1	0	1	1	1	0	0	0	1.9	7
QSC	0	0	0	0	0	2	0	0	1	1	0	0	1.8	8
CRF	0	0	0	0	0	1	0	0	1	2	0	0	1.4	9
SVM	0	0	0	0	0	0	0	0	0	1	3	0	0.8	10
Manifold Ranking	0	0	0	0	0	0	0	0	0	0	1	3	0.4	11

### Complexity analysis of the proposed approach

In this section, the complexity of the proposed approaches both for *with SOM* and *without SOM* based genetic operators are analyzed. Let *N* be the number of solutions, M be the number of objectives to be optimized, *T* be the maximum number of generations.

#### With SOM

1. Population initialization step takes O(N) time as there are *N* solutions which are randomly initialized using a binary vector obeying some constraint. Each solution undergoes objective function calculation step which takes O(NM) time. Thus, the total time complexity of population initialization is O(N+NM) which is equivalent to O(NM).
2. The solutions in the population undergo SOM training which takes $O\left(N^{2}\right)$ time [73].
3. Mating pool generation for each solution takes $O\left(N^{2}\right)$ time as for each solution we have to find neighbors.
4. The time taken for new solution generation using genetic operators (crossover and mutation) is O(N+NM). The term *M* is present because of objective function calculation for each new solution.
5. Evaluation of dominance and the non-dominance relationships between 2*N* solutions (after merging old population and new population) and then the selection of best *N* solutions take $O\left(MN^{2}\right)$ time [14].

Steps-2 to 5 are repeated up to *T* number of generations. Note that updation of SOM training data takes constant time. So it can be ignored. Thus, the total time complexity of the proposed architecture with SOM based operators is

$$\begin{array}{c} O\left(MN+T\left(N^{2}+N^{2}+N+NM+MN^{2}\right)\right). \end{array}$$

On solving further, it gives rise to

$$\begin{array}{c} \Rightarrow O\left(MN+T\left(2N^{2}+NM+MN^{2}\right)\right)\\ \Rightarrow O\left(MN+T\left(MN^{2}\right)\right)\\ \Rightarrow O\left(MN(1+TN)\right)\approx O\left(TMN^{2})\right) \end{array}$$

which is the worst time complexity of our approach when using SOM based genetic operators.

#### Without SOM-based genetic operators

In the proposed architecture without SOM based genetic operators, step-2 and step-3 will not be there. Here, the mating pool for each solution is the entire population. Other steps will remain the same. Thus total time complexity without SOM based genetic operators is

$$\begin{array}{c} O\left(MN+T\left(N+NM+MN^{2}\right)\right)\approx O\left(MN+TMN^{2}\right)\\ \Rightarrow O\left(MN\left(1+TN\right)\right)\approx O\left(TMN^{2}\right) \end{array}$$

which is the same as the time complexity of the proposed architecture when developed with SOM based genetic operators.

## Conclusions and future works

In this paper, an extractive single document text summarization (ESDocSum) system is developed. The key-contributions of the proposed approach are the following: 1) a self-organized multi-objective binary differential evolution based technique is proposed for summary extraction. It utilizes the topological space identified by SOM to develop some new genetic (selection) operators; 2) the similarity/dissimilarity between two sentences is calculated utilizing three measures: normalized google distance, word mover distance and cosine similarity to show that summarization results not only depend on proposed framework but also depend on type of similarity/dissimilarity measures used; 3) six objective functions are utilized for selecting a good subset of sentences present in the document; 4) various unsupervised methods are explored to select a single best summary from the available set of summaries on the final Pareto optimal front; 5) results on standard datasets prove the efficacy of the proposed technique in comparison to state-of-the-art in terms of faster convergence and better ROUGE scores.

Experimental results demonstrate that our SOM-based approach with WMD as a distance measure has obtained 45% and 5% improvements over the best existing method considering ROUGE−2 and ROUGE−1 scores, respectively, for the DUC2001 dataset. While for the DUC2002 dataset, improvements obtained by our approach are 20% and 5%, considering ROUGE−2 and ROUGE−1 scores, respectively. Results are also validated using statistical significance test.

As the performance of summarization system depends on the type of similarity/dissimilarity measure used and also depends on the dataset, therefore, in future, we will try to make the similarity/dissimilarity measure selection automatic for different datasets. In future, we also want to extend the current approach for solving multi-document summarization problem.
